# Integrated Analysis of Terpenoid Profiles and Full-Length Transcriptome Reveals the Central Pathways of Sesquiterpene Biosynthesis in *Atractylodes chinensis* (DC.) Koidz

**DOI:** 10.3390/ijms26031074

**Published:** 2025-01-26

**Authors:** Zheng Zhang, Yelin Tian, Xu Qiao, Hanqiu Li, Lizhi Ouyang, Xinyu Li, Xin Geng, Li Xiao, Yimian Ma, Yuan Li

**Affiliations:** 1 National Engineering Laboratory for Breeding of Endangered Medicinal Materials, Institute of Medicinal Plant Development, Chinese Academy of Medical Sciences & Peking Union Medical College, Beijing 100193, China; 2 School of Landscape Architecture, Beijing University of Agriculture, Beijing 102200, China; 3 Institute of Grain Groups, Xinjiang Academy of Agricultural Sciences, Urumqi 830091, China; 4 Institute of Plant Protection, Chinese Academy of Agricultural Sciences, Beijing 100193, China

**Keywords:** sesquiterpene, eudesmol, transcriptome, terpenoid biosynthesis pathway, *Atractylodes chinensis*, genetic engineering

## Abstract

*Atractylodes chinensis* (DC.) Koidz. is an aromatic and medicinal plant in East Asia. The primary bioactive compounds in this species are sesquiterpenes, particularly β-eudesmol, hinesol, and atractylon. Cultivation techniques require improvement to meet the medicinal demands of this species. In this study, gas chromatography–mass spectrometry analysis of an *A. chinensis* germplasm showed its essential oil contained various sesquiterpenes, including a high relative ratio of β-eudesmol. Full-length transcriptome profiling of *A. chinensis* revealed 26 genes related to terpenoid biosynthesis. These genes belonged to 13 gene families, including five in the isopentenyl pyrophosphate synthase gene family and four in the terpene synthase gene family. The functions of the four terpene synthase genes were proposed based on gene expression patterns and phylogenetic relationships: one was thought to encode monoterpene synthase and three to encode sesquiterpene synthase. Based on the results, the central biosynthesis pathways of the major sesquiterpenes in the *A. chinensis* rhizome were proposed, and three sesquiterpene synthase genes were identified as expressed in the rhizome for the first time. *AcHMGR*, *AcFPPS*, and the three sesquiterpene synthase genes were proposed as potential targets for molecular breeding in *A. chinensis* to enhance its sesquiterpene content.

## 1. Introduction

*Atractylodes chinensis* (DC.) Koidz. is a perennial aromatic plant of the family Asteraceae. The dried rhizome of *A. chinensis* has been widely used in the treatment of rheumatic diseases, digestive disorders, night blindness, and influenza in some East Asian countries, such as China, Korea, and Japan [1]. *A. chinensis* and *A. lancea* are source species of Atractylodes rhizoma, which is commonly known in China as *Cangzhu* and is listed in the Chinese Pharmacopoeia [2]. *A. chinensis* contains various chemical components. Sesquiterpenes are its major medicinal constituents and have various pharmacological activities [3]. In *A. chinensis*, some compounds such as β-eudesmol, hinesol, atractylon, atractylenolide I, atractylenolide III, and atractylodin have been identified as quality markers (Q-markers) that are closely associated with the medicinal efficacy of *A. chinensis* [4,5]. With the exception of atractylodin, which is a polyacetylene, all these components are sesquiterpenes.

In recent years, demand for *Cangzhu* has increased rapidly. With the rapid decrease in wild *A. lancea* resources, *A. chinensis* from northern China has gradually become the main source species of *Cangzhu* provided to the Chinese market. During the outbreak of coronavirus disease 2019 (COVID-19), there was a significant increase in the consumption of *Cangzhu*, which was primarily due to its extensive use in various prescriptions to treat or alleviate symptoms associated with the virus [6]. Nevertheless, the long growth period and low seed-setting rate of *A. chinensis* have hindered the replenishment of *Cangzhu* stocks. The present shortage in *Cangzhu* supply has promoted progress in the cultivation and breeding of *A. chinensis*. However, the variability in *A. chinensis* germplasm resources leads to inconsistencies that significantly affect *Cangzhu* quality. Specifically, cultivated *A. chinensis* generally exhibits reduced levels of medicinal compounds compared to its wild counterpart. To address these issues, new breeding techniques are required.

Recently, molecular breeding has emerged as an important method that is increasingly applied in the breeding of medicinal plants. However, the genome of *A. chinensis* is not publicly available, and there is limited information on the biosynthesis pathways of the sesquiterpene components of *A. chinensis*. The lack of information about the genes involved in the terpenoid biosynthesis pathway has hampered the selection of gene targets for improving the quality of *A. chinensis* via molecular breeding.

Sesquiterpenes are a group of C15 compounds. They represent one of the most prevalent classes of terpenes, which consist of three isoprene building units. In plants, the biosynthesis of sesquiterpenes relies on two key precursors: isopentenyl diphosphate (IPP) and dimethylallyl diphosphate (DMAPP). These precursors come from two pathways: the 2-C-methyl-D-erythritol 4-phosphate (MEP) pathway and the mevalonate (MVA) pathway. While these two pathways are physically separated, they interact at the molecular and metabolic level [7,8]. Some enzymes play significant roles in the two pathways, including 1-deoxy-D-xylulose 5-phosphate synthase (DXS), 1-deoxy-D-xylulose 5-phosphate reductoisomerase (DXR), and hydroxymethylglutaryl-CoA reductase (HMGR). Once IPP and DMAPP are produced, they are utilized by isopentenyl pyrophosphate synthases (IDSs) to generate various carbon skeletons with different isopentenyl diphosphate units. These carbon skeletons then undergo cyclization and rearrangement, ultimately producing carbon skeletons specific to each terpene category via terpene synthases (TPSs). Sesquiterpene synthases (STSs) are the key TPSs involved in the biosynthesis of sesquiterpene. This set of enzymes exhibits a range of sequence similarities but has diverse functions [9].

In recent years, some putative genes related to sesquiterpene biosynthesis in *A. chinensis* have been revealed by Illumina-based transcriptome high-throughput sequencing [10]. However, some challenges remain. First, high-throughput sequencing based on the Illumina platform has limitations in full-length transcript identification. Second, some key enzyme genes involved in sesquiterpene biosynthesis are not conserved and cannot be predicted exactly based only on sequence similarities. Finally, the functions of some gene family members remain unclear.

In this study, an *A. chinensis* germplasm containing a relatively high level of essential oil was chosen as the experimental material. Chemical analysis detected various sesquiterpene constituents in the essential oil extracted from the rhizome of the *A. chinensis* germplasm, of which β-eudesmol had the highest relative content. A full-length transcriptome profiling method (based on the PacBio platform) was used to systematically identify 26 putative full-length genes belonging to 13 gene families related to terpene biosynthesis in the *A. chinensis* germplasm. The functions of some key genes were proposed based on their tissue-specific expression patterns and the phylogenetic classification of the encoded proteins. Several genes emerged as potential gene targets to improve the sesquiterpene content of *A. chinensis* via molecular breeding. Additionally, the proposed biosynthesis pathway for sesquiterpenes in the *A. chinensis* rhizome will contribute to the understanding of the biosynthesis process for some specific plant sesquiterpenes.

## 2. Results

### 2.1. Identification of an A. chinensis Germplasm and Analysis of Its Volatiles

**Figure 1:**
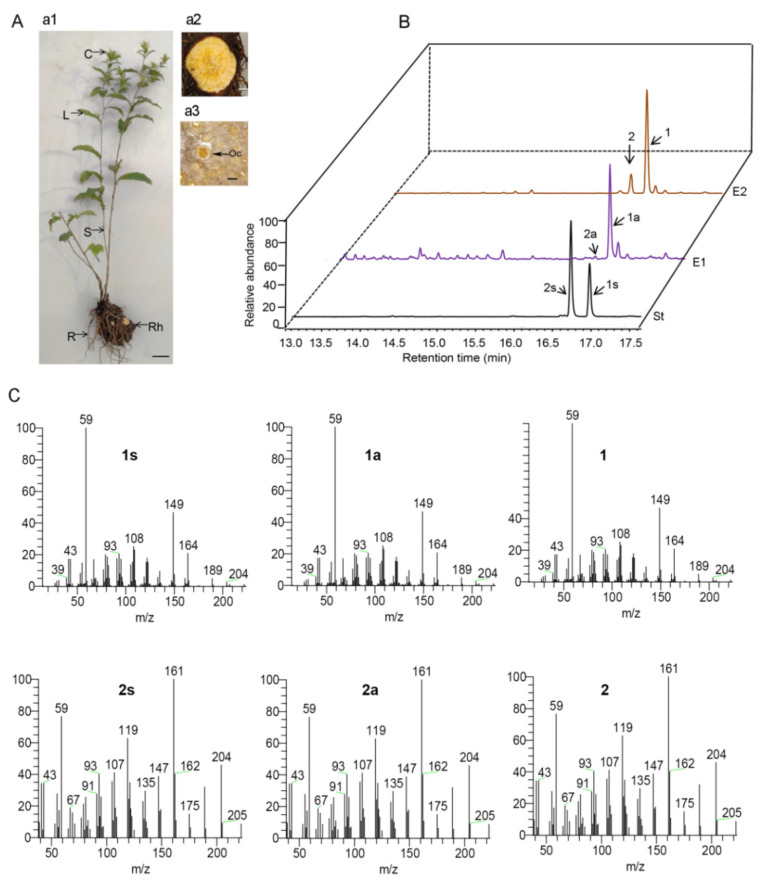
The main sesquiterpenes derived from the rhizome of five-year-old *A. chinensis*. (**A**) The morphological characteristics of *A. chinensis*. C, L, S, R, and Rh in picture a1 represent the capitulum, leaf, stem, root, and rhizome of *A. chinensis*, respectively (scale bar = 3 cm). Picture a2 shows the cross-section of the rhizome under low magnification (scale bar = 2.5 mm). Picture a3 shows the cross-section of the rhizome under high magnification (scale bar = 500 µM; Oc = oil cavity). (**B**) Total ion GC–MS chromatograms for the authentic standards of β-eudesmol and hinesol (St), the volatiles extracted from the rhizome of *A. chinensis* using *n*-hexane (E1), and the essential oil extracted from the rhizome of *A. chinensis* (E2). (**C**) Mass spectra of the sesquiterpenes. 1s, 1a, and 1 represent the mass spectra of the St, E1, and E2 ion peaks at 16.98 min. 2s, 2a, and 2 represent the mass spectra of the St, E1, and E2 ion peaks at 16.78 min.

For this study, some wild *A. chinensis* plants were collected from Chengde City in Hebei Province, China, and cultivated in the medicinal plant garden of the Institute of Medicinal Plant Development, Chinese Academy of Medical Sciences and Peking Union Medical College. From this *A. chinensis* germplasm, a five-year-old plant was selected for morphological and chemical analysis. As shown in Figure 1A, this *A. chinensis* specimen was about 60 cm high with a single erect stem. It had a branched upper part with some white capitula. When observed under the microscope, numerous small reddish-brown oil cavities were observed in the rhizome section (Figure 1A), which suggested a high concentration of essential oil. The essential oil was extracted from the rhizome by the steam distillation method, with an average extraction ratio of 3.33% mL·g^−1^. The volatiles were also extracted from the rhizome using *n*-hexane. Both extracts were analyzed by GC-MS. As shown in the total ion GC-MS chromatogram, the highest peak (16.98 min) and the peak immediately preceding it (16.78) were identified as β-eudesmol and hinesol by comparing their retention time and mass spectra with the authentic standards (Figure 1B,C). The β-eudesmol content in the rhizome of the *A. chinensis* germplasm was finally determined to be 1.51% using high-performance liquid chromatography. This level is higher than that found in most *A. chinensis* rhizome materials we collected in northern China during our previous work [11].

The C_15_ volatiles from the essential oil of the *A. chinensis* germplasm were further identified by comparing the standard mass spectrum data and the RI values in the NIST2017 database. A total of 20 compounds were identified, including some isomers (Table 1 and Figure S1). Among these, 12 were isomers of C_15_H_24_, 5 were isomers of C_15_H_26_O, and the remaining 3 were atractylon (C_15_H_20_O), aristolone (C_15_H_22_O), and atractylodin (C_13_H_10_O). These compounds comprised 19 sesquiterpenes and 1 polyacetylene (atractylodin). Based on the peak heights and areas in the ion chromatogram, the most abundant component was β-eudesmol (45.23%), followed by atractylodin (16.84%), hinesol (8.52%), and atractylon (3.24%). Of these components, β-eudesmol, hinesol, and atractylon are characteristic bioactive sesquiterpenes of *A. chinensis*. Polyacetylene atractylodin is an index component of *A. chinensis* stipulated in the Chinese Pharmacopoeia (2020) [2]. These results indicated that the *A. chinensis* germplasm contained various bioactive ingredients and was a good material for breeding studies.

### 2.2. High-Throughput Sequencing and Functional Gene Prediction

The full-length functional genes of *A. chinensis* were acquired by sequencing a mixture of equal proportions of RNA from the rhizomes of three- and five-year-old plants on a PacBio Sequel platform. More than 22.54 GB of data were generated. The rhizomes of the three- and five-year-old *A. chinensis* were also used for second-generation high-throughput sequencing on an Illumina Novaseq 6000 platform, with about 6 GB of data generated for each sample. The PacBio- and Illumina-based sequencing data were mutually corrected, and redundancies were removed. After the sequences were assembled and processed, a total of 18,118 unigenes were acquired with different length–frequency distributions (Figure S2A). All 18,118 unigenes were searched against public databases for gene function prediction and classification. Searching against the Nr, SwissProt, and Nt databases yielded annotations for 17,731, 15,628, and 14,567 unigenes, respectively. In the KEGG, KOG, and GO databases, 17,606, 11,673, and 13,042 unigenes, respectively, were classified into different categories. Additionally, the conserved domains for 13,042 unigenes were predicted by searching against the Pfam database. In total, annotation information was acquired for 17,805 unigenes by searching at least one database, while information for 8252 unigenes was acquired by searching all seven databases. The number of genes annotated by the seven databases is shown in Figure S2B. After excluding five contaminated sequences, the sequences of all 18,118 assembled unigenes were submitted to the Genbank transcriptome shotgun assembly (TSA) database (accession number GKNN00000000). Then, the clean reads generated by second-generation sequencing of the *A. chinensis* rhizome RNA were mapped on the consensus reference sequences. Using FRKM values > 0.3 as the gene expression threshold, 16,503 and 16,686 unigenes were expressed in the rhizomes of three-year-old (A3) and five-year-old (A5) plants, 15,716 unigenes were expressed in both A3 and A5 (Figure S2C), and 4565 genes were significantly differentially expressed. The distribution and number of the differentially expressed unigenes are shown as a volcano map in Figure S2D.

### 2.3. Prediction of Key Enzyme Genes Involved in the Terpenoid Biosynthesis Pathway

Using the transcriptome data, 26 unigenes that encoded enzymes potentially involved in terpenoid biosynthesis in *A. chinensis* were discovered. All these unigenes had a full open reading frame (ORF) that exceeded 100 amino acids. The 26 genes included seven encoding enzymes in the MEP pathway, nine encoding enzymes in the MVA pathway, one encoding isopentenyl diphosphate isomerase (IDI), five encoding IDSs, and four encoding TPSs. The five genes encoding IDSs were named *AcFPS1*, *AcGGPS1*, *AcSSUII*, *AcPPS*, and *AcSPS* based on the subsequent phylogenetic analysis, and the four terpene synthase genes were named *AcTPS1*, *AcSTS1*, *AcSTS2*, and *AcSTS3*. The sequence characteristics of the putative enzymes are shown in Table S1. These contained 289 to 740 amino acids with isoelectric points from 4.37 to 9.17. According to the YLoc web server prediction, chloroplasts were the localization site for all enzymes in the MEP pathway, whereas the cytoplasm was the predominant localization site for most enzymes in the MVA pathway. The IDI and IDS enzymes were predicted to be in chloroplasts, the cytoplasm, or mitochondria. The putative TPSs were predicted to be in chloroplasts or the cytoplasm. Some enzymes were predicted to be in multiple cellular parts. Reverse-transcription PCR and cDNA sequencing revealed that the coding sequences for ten unigenes were consistent with the predicted sequences, namely, *AcAACT3*, *AcHMGS1*, *AcHMGS3*, *AcHMGR*, *AcPMK*, *AcMDC*, *AcIDI*, *AcTPS1*, *AcSTS1*, and *AcSTS2*.

### 2.4. Phylogenetic Analysis of Enzymes Involved in the MEP and MVA Pathways

Seven full-length unigenes encoding five enzymes involved in the MEP pathway were discovered, including two encoding DXS, two encoding DXR, one encoding 2-C-methyl-D-erythritol 4-phosphate cytidylyltransferase (MCT), one encoding 4-hydroxy-3-methylbut-2-enyl diphosphate synthase (HDS), and one encoding 4-hydroxy-3-methylbut-2-enyl diphosphate reductase (HDR). DXS and DXR play crucial roles in the first stage of terpenoid biosynthesis. DXS catalyzes the synthesis of 1-deoxy-D-xylulose 5-phosphate (DXP) from pyruvate and D-glyceraldehyde 3-phosphate (G3P). The DXS family consists of three separate groups in plants. The DXS1 group is found in tissues that participate in photosynthesis and are necessary for basic functions [12,13]. The DXS2 group is present in plastids and may contribute to producing isoprenoids involved in defense responses and signaling [13]. The DXS3 group may be involved in the production of some hormones [14]. An analysis of plant DXS protein phylogeny indicated that AcDXS1 was part of the DXS1 group and AcDXS2 was part of the DXS2 group (Figure S3A). Both AcDXS1 and AcDXS2 had plastid signal peptides, with strong confidence (Table S1). Sequence alignment revealed AcDXS1 to be highly similar to AlDXS1 (ARB15801.1) from *A. lancea* but with eight amino acid differences (Figure S4). DXR is a key enzyme in the MEP pathway, which transforms DXP into MEP. In *A. chinensis*, two genes encoding AcDXR were discovered. By phylogenetic analysis, both AcDXR1 and AcDXR2 were type I, which are present in the major dicot plants (Figure S3B). Sequence alignment indicated that AcDXR1 and AcDXR2 were highly similar to AlDXR1 (AWH12899) and AlDXR2 (AWH12898) from *A. lancea* but with several amino acid differences (Figure S5).

There were nine full-length unigenes participating in the MVA pathway in *A. chinensis*. As shown in Figure S6A, according to phylogenetic analysis, plant Acetyl-CoA C-acetyltransferase (AACT) is divided into three clusters: a gymnosperm group (cluster III) and two angiosperm groups (clusters II and I). AcAACT1, AcAACT2, and AlAACT belonged to cluster I, which also included AtAACT2—a protein required for embryogenesis and normal male gamete transmission [15]. AcAACT3 belonged to cluster II, which also included AtAACT1 (functionally redundant with AtAACT2). Sequence alignment showed that AcAACT3 had a PTS1 tripeptide (SRL) at the C-terminal, which represented the peroxisome localization signal of AtAACT1 (Figure S7) [16,17]. Phylogenetic analysis showed the three hydroxymethylglutaryl-CoA synthases (HMGRs) in *A. chinensis* were all included in the dicot plant clusters, with AcHMGS2 and AcHMGS3 having > 80% similarity (Figure S6B). It is believed that HMGR acts as a limiting enzyme in the MVA pathway. According to phylogenetic analysis, plant HMGR proteins are divided into three separate clades: dicot plants (I), monocot plants (II), and gymnosperms (III). Clade I could be further divided into two sub-clades, I-a and I-b (Figure S6C). The HMGR proteins from *A. chinensis* (AcHMGR) and *A. lancea* (AlHMGR) were highly similar and belonged to dicot plant group I-a but with three amino acid differences (Figure S8).

### 2.5. Identification of Isopentenyl Pyrophosphate Synthase Genes

**Figure 2:**
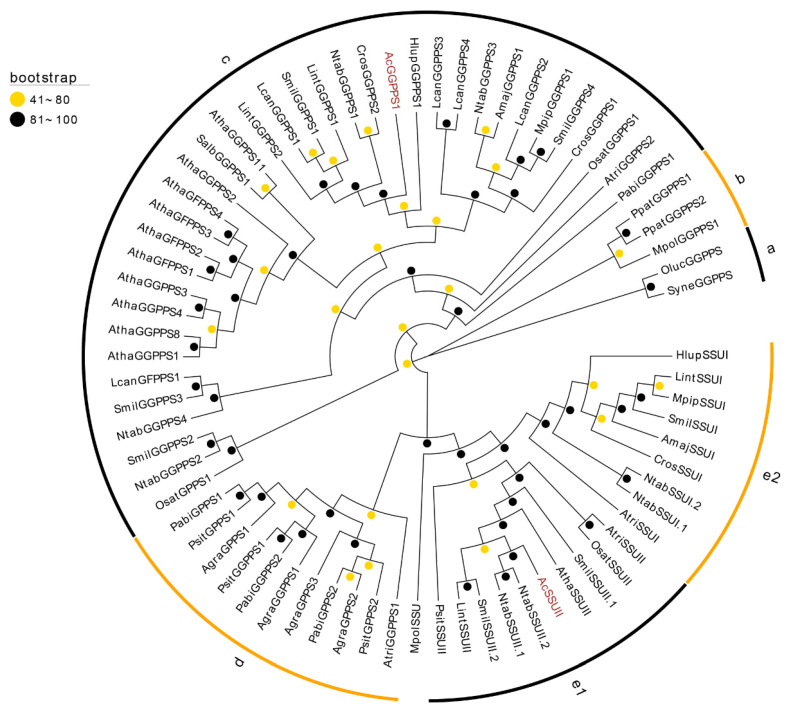
Analysis of the family members of geranylgeranyl diphosphate synthase (GGPPS) using phylogenetic methods. The maximum-likelihood phylogenetic tree was constructed using IQ-Tree with the ultrafast bootstrap (1000 replicates) and the best-fit JTT+R5 models. AcSSUII and AcGGPPS1 are highlighted in brown. The orange and black balls at the nodes represent bootstrap value ranges of 41–80% and 81–100%, respectively. The names of the species are abbreviated as Agra (*Abies grandis*), Amaj (*Antirrhinum majus*), Atha (*Arabidopsis thaliana*), Atri (*Amborella trichopoda*), Cros (*Catharanthus roseus*), Hlup (*Humulus lupulus*), Lcan (*Leucosceptrum canum*), Lint (*Lavandula intermedia*), Mpip (*Mentha piperita*), Mpol (*Marchantia polymorpha*), Ntab (*Nicotiana tabacum*), Oluc (*Ostreococcus lucimarinus*), Osat (*Oryza sativa*), Pabi (*Picea abies*), Ppat (*Physcomitrium patens*), Psit (*Picea sitchensis*), Salb (*Sinapis alba*), Smil (*Salvia miltiorrhiza*), and Syne (*Synechocystis* sp. PCC 6803). The details for all proteins are provided in Table S2.

The present study identified a single gene encoding IDI, which potentially plays a crucial role in the conversion of IPP and DMAPP [18,19]. The following trans-IDSs participate in the production of short-chain isopentenyl pyrophosphate and serve as key metabolic branch points of terpenoid synthesis in plants [20]. Five genes encoding trans-IDSs were identified in this study. Of these, *AcSPS* encodes a protein homologous to chloroplastic-type solanesyl diphosphate synthase (SPS), so it may play a role in the formation of the plastoquinone-9 side chain in plastids [21,22]. *AcPPS* encodes a homolog of AT2G34630, which is a polyisopentenyl pyrophosphate (PPS) required for the biosynthesis of ubiquinone. *AcFPPS* encodes a protein with high similarity to plant farnesyl diphosphate synthase (FPPS). Sequence alignment indicated that AcFPPS contained the conserved FARM and SARM motifs (Figure S9). Therefore, *AcFPPS* is potentially involved in the production of farnesyl diphosphate (FPP). The remaining two IDS genes were named *AcGGPPS1* and *AcSSUII*, and the phylogenetic analysis revealed that these encoded proteins were homologous to GGPPS and the type-II small subunit (SSUII). In plants, geranyl diphosphate synthase (GPPS) or GGPPS usually function as dimers. Although GGPPSs typically form homodimers, GPPSs exist as homo- and heterodimers. The heterodimer-type GPPS usually consists of a GGPPS-homologous small subunit (SSU) and a large subunit (LSU), which is usually a GGPPS [23,24,25,26,27]. Plants have two kinds of SSUs. Type-I SSUs (SSU-I) can produce geranyl diphosphate (GPP), while type-II SSUs (SSU-II) can interact with LSU to produce GPP and can also increase the production of geranylgeranyl diphosphate (GGPP). A phylogenetic tree of plant IDSs was constructed (Figure 2). The IDS members were classified into five clades, with clade e representing the SSU group, which was further classified into groups e1 (SSUII) and e2 (SSUI). AcGGPPS1 was clustered into clade c, representing the group with plant GGPPSs and geranylfarnesyl diphosphate synthases (GFPPSs) and which included AtGGPPS11_LSU (AT4G36810). AcSSUII was clustered into group e1, which represented the type-II SSUs. To further analyze their sequence characteristics, the AcSSUII and AcGGPPS1 sequences were aligned with other plant homologs. The specific structures, such as FARM and SARM, and the specific motifs are shown in Figures S10 and S11.

### 2.6. The Phylogenetic Relationships of AcTPS1 and AcSTSs with Other Plant Terpene Synthases

According to the phylogenetic relationships, plant TPS can be categorized into seven groups: TPS-a, -b, -c, -d, -e/f, -g, and -h [28,29]. In most angiosperms, the TPS-a and TPS-b groups mainly contain sesquiterpenes and monoterpenes. In this study, four putative TPS genes were identified in the assembled unigenes: *AcTPS1*, *AcSTS1*, *AcSTS2*, and *AcSTS3*. These sequences were submitted to Genbank and assigned the accession numbers OR514633, OR514634, OR514635, and OR514636, respectively. According to their coding sequences, the encoding proteins were predicted to be AcTPS1 and AcSTS1–3. Searching against the NCBI Nr database revealed AcTPS1 as having 72% similarity (query coverage = 99%; E-value = 0) with a putative chloroplastic-type R-linalool synthase QH1 in *Lactuca sativa* (query coverage = 99%; E-value = 0). AcSTS1, AcSTS2, and AsSTS3 showed high similarities with some other plant STSs.

**Figure 3:**
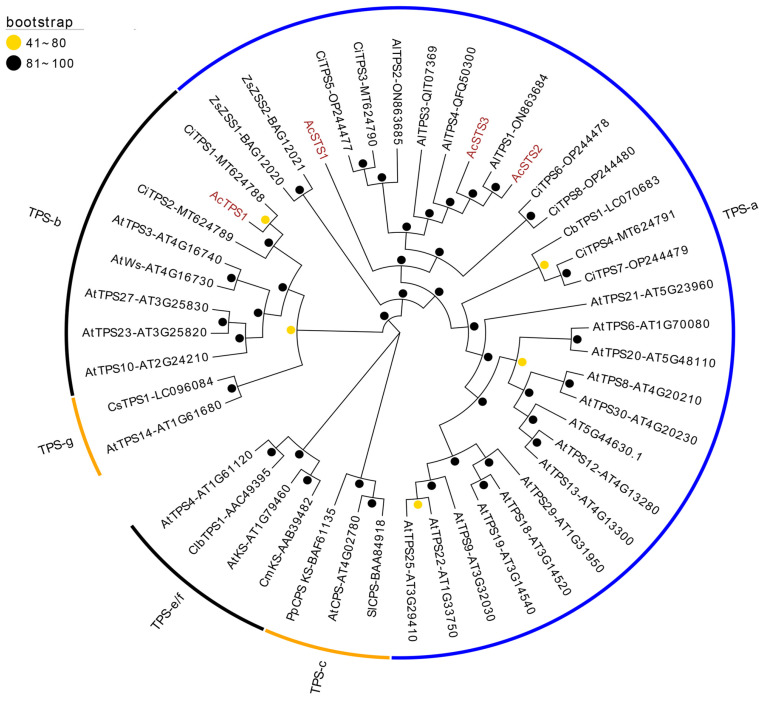
The phylogenetic relationships of terpene synthases between *A. chinensis* and some other plant species. The unrooted phylogenetic tree was constructed using the IQ-TREE 2 package with 1000 bootstrap replicates and modified using the online EvolView tool (version 2). The putative *A. chinensis* terpene synthase proteins are highlighted in brown. As the legend shows, the orange and black balls at the nodes represent bootstrap values 41–80% and 81–100%, respectively. The enzymes are named using species abbreviations as prefixes, such as At (*Arabidopsis thaliana*), Ac (*A. chinensis*), Al (*A. lancea*), Pp (*Physcomitrella patens*), Cb (*Camellia brevistyla*), Cs (*Camellia saluenensis*), Ci (*Chrysanthemum indicum*), Cm (*Cucurbita maxima*), Zs (*Zingiber zerumbet* Smith), and Clb (*Clarkia breweri*).

According to the sequence alignment result, all putative *A. chinensis* TPSs contained key motifs, such as RRx8W, RxR, DDxxD, and NSE/DTE (Figure S12). A phylogenetic tree was constructed to analyze their relationships with some other plant species (Figure 3). The results showed that AcTPS1 belonged to the TPS-b group and was a close relative of CiTPS1 (MT624788) from *Chrysanthemum indicum*. Because it has been proven that the recombinant proteins of CiTPS1 produce α-pinene, which is a monoterpene [30], it is suggested that AcTPS1 may be involved in the production of monoterpene. AcSTS1, AcSTS2, and AcSTS3 belonged to the TPS-a group, which mainly includes plant STSs. In the three putative STSs, AcSTS2 and AcSTS3 were clustered with AlTPS1, which is from *A. lancea* and can catalyze the in vitro production of elemol [31]. AcSTS1 showed a close relationship with CiTPS6 and CiTPS8. In vitro activity analysis has shown that STSs CiTPS6 and CiTPS8 can produce multiple products (such as δ-cadinene, germacrene-4-ol, and α-cadinol) when FPP is the substrate [32]. So, it is suggested that AcSTS1, AcSTS2, and AcSTS3 may be responsible for the production of multiple sesquiterpenes in *A. chinensis*.

### 2.7. Analysis of Tissue-Specific Gene Expression Patterns

**Figure 4:**
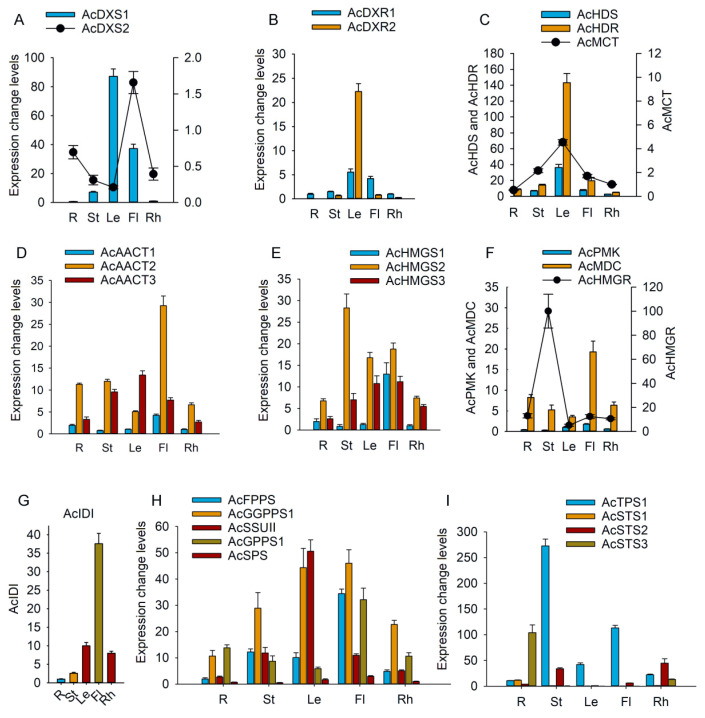
Tissue expression patterns of enzyme genes involved in terpenoid biosynthesis pathways in *A. chinensis*. R, St, Le, Fl, and Rh represent root, stem, leaf, capitulum (flower), and rhizome, respectively. The y-axis represents fold changes in gene expression levels. To make it easier to compare the changes, a two-y-axis graph was used to display the expression changes for certain genes. Graphs A, C, and F demonstrate this by showing different amplitudes of expression changes using two y-axes, with the specific genes indicated. To exhibit the gene expression fold changes in (**A**–**E**,**H**,**I**), the expression levels of *AcDXS1*, *AcDXR1*, *AcMCT*, *AcAACT1*, *AcHMGS1*, *AcSPS*, and *AcSTS1* in rhizomes were arbitrarily set to 1. The expression levels of *AcPMK* in leaves and *AcIDI* in roots were set to 1 in (**F**) and (**G**), respectively.

The tissue-specific expression patterns of these genes were analyzed by real-time quantitative PCR (qPCR). In the MEP pathway, among the three *AcDXS* genes, *AcDXS1* exhibited higher expression levels than *AcDXS2* in various tissues. *AcDXS1* was expressed most highly in leaves, while *AcDXS2* was expressed most highly in flowers (Figure 4A). This indicated that *AcDXS1* might perform a role in the production of terpenoids in leaves, whereas *AcDXS2* could be associated with the synthesis of particular terpenoids in flowers. Both *AcDXR1* and *AcDXR2* exhibited the highest expression levels in leaves (Figure 4B), as did *AcHDS*, *AcHDR,* and *AcMCT*. In various tissues, *AcMCT* had lower expression levels than *AcHDS* and *AcHDR* (Figure 4C). These results indicated that the MEP-pathway genes in *A. chinensis* are active primarily in photosynthetic tissues.

In the MVA pathway, among the three *AcAACT* genes, *AcAACT2* exhibited the highest expression levels in flowers (Figure 4D). This finding supported its role deduced from the phylogenetic analysis, as related to embryogenesis and normal male gamete transmission. *AcAACT3* exhibited the highest expression level in leaves. Among the three *AcHMGS* genes, *AcHMGS2* showed the highest expression in stems, followed by flowers and leaves (Figure 4E). *AcHMGR* also displayed the greatest expression level in stems. Compared with *AcHMGR*, *AcPMK* and *AcMDC* exhibited lower expression levels in various tissues and the highest expression level in flowers (Figure 4F). It has been reported that the *HMGR* genes of *Glycine max* regulate its isoprenoid biosynthesis [33]. Abscisic acid regulates *PqHMGR* gene expression and triterpenoid saponin production in hairy root cultures of *Panax quinquefolium* [34]. So, *AcHMGR* is a putative metabolic engineering target that can be used to improve the sesquiterpenoid yield of *A. chinensis*.

Among the genes encoding enzymes at the following stages, *AcIDI* exhibited higher expression in flowers than in other tissues (Figure 4G). The *AcIDS* genes exhibited various expression patterns (Figure 4H). *AcFPPS* was a key enzyme in sesquiterpene biosynthesis. It was expressed most strongly in flowers, with lower expression levels in leaves, stems, rhizomes, and roots. Among the other *IDS* genes, *AcSPS* displayed the most significant expression in leaves, indicating its role in the synthesis of plastoquinone-9 within plastids. *AcPPS*, which is probably involved in ubiquinone side-chain biosynthesis, exhibited the highest expression level in flowers. The expression patterns of *AcSSUII* and *AcGGPPS1*, which produce proteins that potentially constitute the small and large subunits of heterodimeric IDSs and participate in the biosynthesis of monoterpene or diterpene, showed different expression trends. In the root, stem, flower, and rhizome, the expression of *AcGGPPS1* was higher than that of *AcSSUII*; in leaves, *AcSSUII* exhibited higher expression levels than *AcGGPPS1*. Based on the expression patterns of the two genes and prior studies on heterodimer GPPSs [25,35], it is suggested that the main function of AcSSUII is not to produce GPP but to increase the production of GGPP through interacting with AcGGPPS1 in the leaves. In the four genes encoding the TPSs, *AcTPS1* exhibited the highest expression levels in stems. *AcSTS1* and *AcSTS2* exhibited the highest expression levels in roots, while *AcSTS2* exhibited the highest expression level in rhizomes (Figure 4I). This indicated that the three *AcSTS* genes may play different roles in sesquiterpene synthesis in *A. chinensis*.

## 3. Discussion

### 3.1. Identification of the Sesquiterpenes in A. chinensis Germplasm with High Levels of Essential Oil

In this study, an *A. chinensis* germplasm was determined to have >3% (*v*/*w*) essential oil in its rhizome, which is higher than the average content. Microscopic observation showed many oil cavities in the rhizomes, which are the major structures for storing essential oils. GC-MS detected 19 kinds of sesquiterpenes and 1 polyacetylene in its essential oil (Table 1). These included β-eudesmol, with the highest relative content (45.23%), followed by atractylodin (16.84%), hinesol (8.52%), and atractylon (3.24%). The content of β-eudesmol was estimated to be over 1.5% of the total weight of the rhizome, which is regarded as a comparably higher level based on our previous work [11]. Since β-eudesmol, hinesol, atractylon, and atractylodin are potential Q-markers of *A. chinensis* [4,5], this *A. chinensis* germplasm was judged to be of high quality and useful for the cultivation and breeding of *A. chinensis*.

Because the essential oil extracted from the rhizome of this *A. chinensis* germplasm contained high levels of bioactive sesquiterpenes, it could be used as a natural antioxidant and antibacterial agent with potential applications in the food and pharmaceutical industries. β-Eudesmol, found in essential oils, has numerous bioactive properties, including anti-tumor, anti-angiogenic, anti-inflammatory, and anti-allergic effects [36]. Apart from *A. chinensis*, β-eudesmol is also present in *Humulus lupulus* (hop), *Magnolia officinalis*, and *Zingiber zerumbet* [37,38,39]. Notably, however, only the essential oil from *A. chinensis* contains a substantial amount of β-eudesmol without its isomer α-eudesmol. Therefore, essential oil derived from this *A. chinensis* germplasm would be valuable for medicinal research due to its high concentration of β-eudesmol. The essential oil derived from the *A. chinensis* germplasm also contained other sesquiterpenes, such as hinesol, β-selinene, and elemol. These substances give the oil the potential to be used as a natural insecticide [40]. Additionally, the essential oil from this *A. chinensis* contained a relatively high level of atractylodin, which has various pharmacological activities, such as anti-colitis [41,42].

### 3.2. Identification of Full-Length Genes Involved in Terpenoid Biosynthesis Pathways

Recently, the combination of volatile terpenoid profiling and transcriptomic analysis has been increasingly used to uncover the functional genes associated with terpene biosynthesis. For example, using this approach, the terpenoid profiles of *Chrysanthemum morifolium* and *C. indicum* were revealed [30,32,43]. In the present study, chemical and transcriptomic analysis revealed 26 full-length enzyme genes related to the terpenoid biosynthesis in *A. chinensis*. Molecular phylogenetic analysis combined with tissue-specific expression profiling suggested that *AcDXS1* and *AcDXS2* may be mainly involved in terpenoid biosynthesis in leaves and flowers, respectively. *AcDXR1* is likely associated with a housekeeping function, while *AcDXR2* may be related to defense functions. qPCR analysis of tissue-specific expression also suggested that most genes involved in the MEP pathway in *A. chinensis* were active in either leaves or flowers. Consistent with this, the predicted cellular locations of their encoded MEP pathway enzymes were mostly in chloroplasts or plastids.

Although most enzyme genes related to the MEP pathway showed significant expression in leaves and flowers, the expression patterns of the MVA-pathway genes were different. In the key enzymes for sesquiterpene or triterpene production, *AcHMGS2* and *AcHMGR* displayed the highest expression in stems, indicating the significance of stems in sesquiterpene or triterpene synthesis. According to the analysis of tissue-specific expression patterns, most genes involved in the MEP and MVA pathways were expressed at high levels in leaves, flowers, and stems during flowering. This may have indicated that during the flowering stage, *A. chinensis* specifically allocates resources to the production of terpenoids crucial for photosynthesis and flowering, for example, to synthesize the floral scents constituted by monoterpenes and sesquiterpenes to attract pollinators or to deter florivores. The result also suggests that the flowering period may not be an optimal time for harvesting *A. chinensis* rhizomes, as sesquiterpene production in the rhizome is relatively inactive during this phase.

In recent years, trans-IDSs have attracted increasing research interest. Members of the trans-IDS families have been identified in various plant species, including Arabidopsis [44,45], *Andrographis paniculate* [46], Lavandula [47], and *Cinnamomum camphora* [48]. The present study identified five IDS genes in *A. chinensis* and predicted their possible roles in terpenoid precursor biosynthesis, providing a basis for the exploration of the diversity of terpenoids in *A. chinensis*.

Sesquiterpenes, including β-eudesmol, hinesol, and atractylon, are the primary bioactive constituents of *A. chinensis*. These compounds were primarily found in the essential oil extracted from the rhizome of *A. chinensis*. In the present study, four full-length TPS genes in *A. chinensis* were revealed, of which three encoded STS. Phylogenetic analysis revealed that AcSTS2 and AcSTS3 clustered with AlTPS1 (which has been validated to catalyze elemol biosynthesis). In the rhizome, *AcSTS2* exhibited the highest expression level, followed by *AcSTS3*. This suggested that *AcSTS2* and *AcSTS3* play crucial roles in the biosynthesis of sesquiterpenes in the rhizome of *A. chinensis*. In stems, *AcSTS2* exhibited the highest expression level. In roots, *AcSTS1* and *AcSTS3* exhibited higher expression levels than *AcSTS2*. These results indicated that the three *AcSTS* genes play different roles in sesquiterpene biosynthesis in various tissues.

### 3.3. Analysis of the Key Gene Targets in the Central Sesquiterpene Biosynthesis Pathway

Nineteen sesquiterpenes were detected in the essential oil extracted from *A. chinensis* rhizome via the GC-MS method and 29 full-length genes involved in biosynthesis pathways were revealed through transcriptome high-throughput sequencing. The tissue-specific expression patterns of the 29 genes and the phylogenetic relationships of some key enzymes were investigated. Based on these results, the main pathway of sesquiterpene biosynthesis in the *A. chinensis* germplasm was proposed.

**Figure 5:**
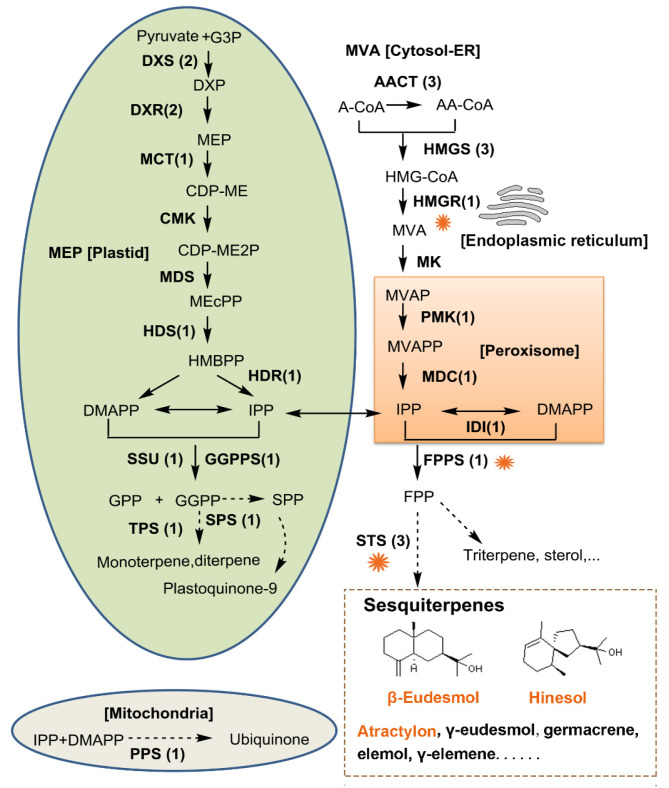
The proposed main pathway of terpene biosynthesis in *A. chinensis*. Enzymes are named on the pathways with the numbers of the enzyme genes in brackets. Except for the abbreviations of enzyme names already indicated in the text, the additional enzyme abbreviations include CMK [4-(cytidine-5′-diphospho)-2-C-methyl-D-erythritol kinase], MDS (2-C-methyl-D-erythritol 2,4-cyclodiphosphate synthase), MK (mevalonate kinase), PMK (5-phosphomevalonate kinase), and MDC (mevalonate pyrophosphate decarboxylase). Except for the abbreviations of compounds already indicated in the text, the additional compound abbreviations include CDP-ME [4-(cytidine 5′-diphospho)-2-C-methyl-D-erythritol], CDP-ME2P [2-phospho-4-(cytidine 5′-diphospho)-2-C-methyl-D-erythritol], MEcPP (2-C-methyl-D-erythritol 2,4-cyclodiphosphate), HMBPP (4-hydroxy-3-methylbut-2-enyl diphosphate), A-CoA (acetyl-CoA), AA-CoA (acetoacetyl-CoA), HMG-CoA (3-hydroxy-3-methylglutaryl-CoA), MVAP (mevalonate-5-phosphate), and MVAPP (mevalonate-5-diphosphate). The key sesquiterpene biosynthesis enzymes are noted with the orange flower symbol. The bioactive sesquiterpenes β-eudesmol, hinesol, and atractylon are highlighted in orange.

As shown in Figure 5, in *A. chinensis* rhizome, sesquiterpenes were mainly synthesized through the MVA pathway. However, the MEP pathway also contributed to this process, as there is cross-talk between MVA and MEP in various plants [49,50,51]. It has been demonstrated that HMGR plays a significant role in regulating terpenoid biosynthesis in various plants, such as *Panax quinquefolium*, *Taraxacum brevicorniculatum*, and *Glycine max* [33,34,52]. Here, it is suggested that HMGR is the first key enzyme in sesquiterpene biosynthesis because it catalyzes the first committed step in the MVA pathway. The next crucial enzyme in the sesquiterpene biosynthesis was FPPS, which catalyzed the production of FPP. Consistent with this, Sun et al. have suggested that the synthesis of polyacetylene atractylodin is closely related to acetyl-CoA carboxylase (ACC), whereas the synthesis of sesquiterpenes atractylenolide II, eudesmol, and atractylon is closely related to HMGR and FPPS [53]. In plants, STS can produce a variety of the core terpene structures of sesquiterpenes. So far, the functions of genes encoding STSs in *A. chinensis* have not been fully elucidated. The present study identified three full-length *AcSTS* genes based on the transcriptome data of *A. chinensis* for the first time and inferred their functions through systematic evolution and tissue-specific analysis.

In recent years, many studies have focused on enhancing terpenoid production through the genetic engineering of key enzyme genes involved in terpenoid biosynthesis. For example, it has been reported that the overexpression of HMGR in *Salvia miltiorrhiza*, *Populus trichocarpa* and *Glycine max* enhanced terpenoid production [33,54,55]. Overexpression of DXS-FPPS and STS in tomato plastids yielded high levels of sesquiterpenes in the fruit [56]. Additionally, overexpression of TPS1 in cotton negatively affected multiple pests while attracting parasitoids [57]. In this study, a series of genes involved in terpenoid biosynthesis in *A. chinensis* was identified. All these genes are potential target sites for molecular breeding of *A. chinensis*. Based on the previous reports, *AcHMGR*, *AcFPPS*, and the three *AcSTS* genes are highly promising as gene targets to improve the sesquiterpene content of *A. chinensis*. In recent years, Wu et al. have summarized the molecular breeding strategies of medicinal plants, such as molecular-marker-assisted breeding, transgene breeding, Sijie breeding, and molecular-design breeding [58]. In our view, the genes identified in this study will be very useful for implementing these strategies to advance molecular breeding of *A. chinensis* in the future.

## 4. Materials and Methods

### 4.1. Plant Materials

The wild resources of *A. chinensis* were collected from the hills of Chengde City, Hebei Province, China. These *A. chinensis* plants were transferred from their natural habitat to the medicinal plant garden of the Institute of Medicinal Plant Development, Chinese Academy of Medical Sciences and Peking Union Medical College. The voucher sample was identified by Professor Zheng Zhang from our group and deposited in the herbarium of our institute. The rhizomes of three- and five-year-old *A. chinensis* plants were sent for high-throughput sequencing. During the flowering period, the capitula, leaves, stems, rhizomes, and roots of a five-year-old *A. chinensis* plant were gathered, enveloped in aluminum foil, and preserved in liquid nitrogen until used for qPCR.

### 4.2. Extraction of the Volatiles from the Rhizome Using n-Hexane

Briefly, the rhizome of the five-year-old *A. chinensis* was dried at room temperature and pulverized using a vibrating mill. A powdered sample of about 1.0 g was sieved through a bore diameter of 0.425 mm. The powder was then extracted with *n*-hexane (10 mL) with the assistance of ultrasonic vibration (pulse energy = 60 kHz) for 30 min at 25 °C. The supernatant was separated by centrifugation for 10 min at 4500× *g* at room temperature and dissolved in *n*-hexane in a ratio of 1:100 for GC-MS analysis.

### 4.3. Extraction of Essential Oil from the Rhizome

First, about 30 g of fresh rhizome from five-year-old *A. chinensis* was cut into small pieces and ground into powder. This powder was soaked in 300 mL water in a steam distillation extractor and then subjected to steam distillation for 8 h. This extraction was repeated three times, and the extraction ratio was determined using the following formula: extraction ratio = average extraction volume/total weight. Finally, the extracted oil was dried with anhydrous sodium sulfate, and a portion of the oil was analyzed by GC-MS.

### 4.4. Gas Chromatography–Mass Spectrometry (GC-MS) Analysis

The volatile constituents extracted from the rhizome of *A. chinensis* and the essential oil were analyzed by GC–MS (Trace 1310–ISQ 7000, Thermo Scientific, Waltham, MA, USA) with the flow rate of carrier gas helium at 1 mL·min^−1^. Each sample aliquot (1 μL) was injected in the split mode (10:1) at 250 °C. The temperature program parameters consisted of 2 min at 50 °C, followed by an increase to 160 °C at a rate of 10 °C·min^−1^ (11 min), then a further increase to 290 °C at a rate of 5 °C·min^−1^ (26 min). The full scan range was 50–500 amu. β-Eudesmol and hinesol were identified by comparison with the retention time and mass spectra of the respective standards purchased from Sigma-Aldrich (St. Louis, MO, USA). The other volatiles were identified by matching their mass spectra with those stored in the NIST 2017 library for GC-MS. Compounds with a similarity of more than 80% were chosen for further analysis. The retention indices (RIs) of the components were determined using a homologous series of n-alkanes (C_8_–C_20_) under the same operating conditions. These RI values were then compared with the RI values in the NIST 2017 library, with the threshold of the relative deviation of the RI values set to 3%.

### 4.5. High-Performance Liquid Chromatography

High-performance liquid chromatography was performed following a method that was previously described [11]. Briefly, the instrument used was a Waters e2695 high-performance liquid chromatography systems (Waters, Milford, MA, USA) with a diode-array detector (Waters 2998). Chromatographic separation was performed on an Agela Venusil MP C18 column (4.6 × 150 mm, 5 μm, 150 A) at 28 °C. The detection wavelength for β-eudesmol was 200 nm. The mobile phase used was acetonitrile: 0.2% NaHCO_3_: 10% methanol (65:25:10). Peak separation was performed over a 30 min isocratic elution at a flowrate of 1 mL/min. The equation for the calibration curve was y = 2E + 07x + 23921 (R^2^ = 0.9999), where x represents the concentration of the sample and y represents the peak area.

### 4.6. High-Throughput Sequencing Based on the PacBio and Illumina Platforms

First, total RNA was extracted from the rhizomes of three- and five-year-old *A. chinensis* using an RNAprep Pure Plant Kit (Tiangen, Beijing, China). Equal quantities of the extracted RNA were mixed and used to produce cDNA according to the SMARTer^®^ PCR cDNA Synthesis Kit protocol (Clontech, San Jose, CA, USA). Subsequently, a library of the mixed sample was constructed and used for single-molecule real-time (SMRT) sequencing on a PacBio Sequel platform (Pacific Biosciences of California, Menlo Park, CA, USA), resulting in the generation of 22.54 GB polymerase read bases. Additionally, an Illumina Novaseq 6000 platform (Illumina, San Diego, CA, USA) was used to conduct second-generation high-throughput sequencing using the RNA extracted from the rhizomes of three- and five-year-old *A. chinensis* (A3 and A5). The raw data of third-generation sequencing were processed using PacBio’s official software package SMRT Link version 5.0 to acquire polished consensus sequences, which were corrected using the Illumina-based sequencing data using LoRDEC [59]. The redundancies of the clustered unigenes were removed by CD-HIT [60]. The transcriptome raw data of *A. chinensis* were submitted to the NCBI SRA database with the following accession numbers: SRR17593274 (the mixed sample sequenced on the PacBio platform), and SRR17593273 and SRR17593272 (samples A3 and A5, respectively, sequenced on the Illumina platform). The assembled unigene sequences, which were generated based on the second- and third-generation sequencing data, were submitted to the NCBI TSA database and arranged with the accession number GKNN00000000.

### 4.7. Gene Prediction and Annotation

The unigenes were searched against the NCBI non-redundant protein sequences (Nr), NCBI nucleotide sequences (Nt), Protein family (Pfam), Clusters of Orthologous Groups of proteins (COG)/euKaryotic Ortholog Groups (KOG), Swiss-Prot, Kyoto Encyclopedia of Genes and Genomes (KEGG), and Gene Ontology (GO) databases. The predicted and annotated functions of the unigenes were determined through analysis of the conserved domains and functional categories, as well as the functional annotations of the homologous genes.

### 4.8. Unveiling the Genes Encoding Enzymes Related to Terpene Production

Two methods were used to search for the key enzyme genes involved in terpene biosynthesis in *A. chinensis* from the assembled unigenes. The first involved searching for the gene annotation results using specific terms, while the second involved a homologous protein search using the basic local alignment search tool (BLAST) [61]. Next, the duplications and non-coding or incomplete sequences, like sequences lacking a full open reading frame (ORF) exceeding 100 amino acids, were eliminated. The resulting genes underwent additional manual verification. The putative protein molecular weights (MWs) and isoelectric points (pIs) were determined using the ExPASy portal online tool (https://web.expasy.org/compute_pi/, accessed on 1 September 2021). The subcellular location of the putative proteins was predicted via the interpretable web server YLoc [62].

### 4.9. Phylogenetic Analysis

Proteins were aligned using the MUSCLE program. The resulting alignment file was used to create a maximum likelihood (ML) phylogenetic tree with 1000 bootstrap replicates using the IQ-TREE version 2.0, as described by Minh et al. (2020) [63]. The best-fit models selected according to BIC were LG+R4 for the DXS tree, JTTDCMut+G4 for the DXR tree, LG+G4 for the AACT tree and HMGS tree, JTTDCMut+R4 for the HMGR tree, JTT+F+R4 for the terpene synthase tree, and JTT+R5 for the IDS tree. The terpene synthase tree and IDS tree were shown as circular cladograms with radial branches representing the clusters, and the other trees were shown as rectangular phylograms. The created tree files were modified using Evolview version 2.0 [64].

### 4.10. Real-Time Quantitative PCR

RNAprep Pure Plant Kits (Tiangen, Beijing, China) were used to extract total RNA from leaves, stems, capitula, roots, and rhizomes. The purity and concentration of the RNA samples were assessed using a NanoDrop 2000 spectrophotometer (Thermo Fisher Scientific, DE, USA). Additionally, the integrity of the RNA was determined by agarose gel electrophoresis. To synthesize first-strand cDNA, 1 μg of total RNA was used following the TIAN Script II M-MLV Reverse Transcriptase protocol (Tiangen, Beijing, China). Real-time quantitative PCR (qPCR) was conducted on a Roche Light Cycler 96 Real-Time System using Trans Start Top Green qPCR Supermix (Transgene, Beijing, China). A melting curve was produced to assess the specificity of the qPCR products. The expression levels were evaluated using the method described by Livak and Schmittgen [65], with *AsUBQ* as the internal reference gene. Gene-specific primers for qPCR were designed with the assistance of Vector NTI 11.0 software (Table S3).

## 5. Conclusions

Because *A. chinensis* is a valuable medicinal resource with increasing demand, improving its cultivation and breeding techniques is crucial. The present study identified the sesquiterpenes of an *A. chinensis* germplasm characterized by a high level of essential oil. Through various methods, such as full-length transcriptome analysis, phylogenetic analysis, and qPCR, the functions of 26 full-length enzyme genes were revealed. Among these, *AcHMGR*, *AcFPPS*, and the three *AcSTS* genes are regarded as the most important gene targets for enhancing sesquiterpene content. This study is the first to provide full-length transcriptome data for the rhizome of an *A. chinensis* germplasm, which provides a reference database for analyzing the molecular mechanisms of *A. chinensis* development and secondary metabolism. Additionally, it provides a significant breakthrough in uncovering the key enzyme genes responsible for the biosynthesis of the major sesquiterpenes in the essential oil of *A. chinensis*. Our future work will focus on the regulatory mechanism of these key enzyme genes, which will facilitate their manipulation for effective molecular breeding strategies.

## Figures and Tables

**Table 1:** Sesquiterpene constituents in the essential oil from *A. chinensis* rhizome.

Peak	^a^ RT	Compound	Formula	CAS	^b^ RI	^c^ RI	^d^ R (%)	Area%
1	12.96	Modephene	C_15_H_24_	68269-87-4	1406	1389	1.22	0.1
2	13.05	Berkheyaradulene	C_15_H_24_	65372-78-3	1413	1392	1.48	0.22
3	13.34	β-Isocomene	C_15_H_24_	71596-72-0	1434	1412	1.56	0.2
4	13.61	γ-Elemene	C_15_H_24_	29873-99-2	1454	1465	0.75	0.52
5	13.96	Humulene	C_15_H_24_	6753-98-6	1480	1473	0.48	0.15
6	14.23	β-Curcumene	C_15_H_24_	28976-67-2	1500	1512	0.79	0.15
7	14.29	α-Muurolene	C_15_H_24_	483-75-0	1504	1511	0.46	0.15
8	14.44	β-Selinene	C_15_H_24_	17066-67-0	1514	1509	0.34	0.33
9	14.69	δ-Cadinene	C_15_H_24_	483-76-1	1531	1521	0.66	0.1
10	14.88	β-Sesquiphellandrene	C_15_H_24_	20307-83-9	1544	1529	0.98	0.46
11	15.15	Naphthalene	C_15_H_24_	58893-88-2	1562	1544	1.18	0.37
12	15.3	Elemol	C_15_H_26_O	639-99-6	1572	1557	0.98	0.87
13	15.51	Germacrene B	C_15_H_24_	15423-57-1	1586	1578	0.54	1.36
14	16.37	Epicubenol	C_15_H_26_O	19912-67-5	1641	1629	0.75	0.15
15	16.64	γ-Eudesmol	C_15_H_26_O	1209-71-8	1658	1635	1.41	1.51
16	16.78	* Hinesol	C_15_H_26_O	23811-08-7	1667	1642	1.51	8.52
17	16.98	* β-Eudesmol	C_15_H_26_O	473-15-4	1679	1657	1.35	45.23
18	17.08	Atractylon	C_15_H_20_O	6989-21-5	1686	1673	0.75	3.24
19	18.35	Aristolone	C_15_H_22_O	6831-17-0	1762	1752	0.54	0.29
20	18.88	Atractylodin	C_13_H_10_O	55290-63-6	1793	1786	0.39	16.84

^a^ RT: retention time for each compound (min). ^b^ RI: retention index, determined on a DB-5 MS column using a homologous series of *n*-alkanes. ^c^ RI: retrieved value of the retention index from NIST 17 database. ^d^ R: |(^b^RI-^c^RI)/^c^RI| × 100%. The relative deviation of the retention index (threshold = 3%). * Represents β-eudesmol being identified using an authentic standard.

## Data Availability

The raw data of the full-length transcriptome of *A. chinensis* based on the Pacbio platform are available in the NCBI SRA database with the accession number SRR17593274. The transcriptome raw data of *A. chinensis* rhizomes based on the Illumina platform are available in the NCBI SRA database with the accession numbers SRR17593273 (A3) and SRR17593272 (A5). The assembled unigene sequences are available in the NCBI transcriptome shotgun assembly (TSA) database with the accession number GKNN00000000. The gene and peptide sequences described in this article can be found in the tables in the Supplementary Materials. The Genbank accession numbers for the four putative terpene synthase genes in *A. chinensis* are OR514633 for *AcTPS1*, OR514634 for *AcSTS1*, OR514635 for *AcSTS2*, and OR514636 for *AcSTS3*.

## Supplementary Materials

The supporting information can be downloaded at: https://www.mdpi.com/article/10.3390/ijms26031074/s1.
