# Supplementary material for: Integrated Analysis of Terpenoid Profiles and Full-Length Transcriptome Reveals the Central Pathways of Sesquiterpene Biosynthesis in Atractylodes chinensis (DC.) Koidz
Source: Int J Mol Sci. 2025 Jan 26;26(3):1074. doi: 10.3390/ijms26031074 (PMC11818032; doi:10.3390/ijms26031074)
Supplement: Supplementary file 1 [file ijms-26-01074-s001.zip › ijms-3342653-supplementary.pdf]

Tab. S1 Characteristics of the putative enzymes in *Atractylodes chinensis* (DC.) Koidz. involved in terpenoid biosynthesis

Enzyme name	EC number[a]	length	MW (kD)	pI	Yloc:Confidence[b]
AcDXS1	EC:2.2.1.7	716	77.06	7	Chloroplast: very strong (0.97)
AcDXS2	EC:2.2.1.7	718	76.97	7.99	Chloroplast:strong (0.89)
AcDXR1	EC:1.1.1.267	473	51.19	6.7	Chloroplast:normal (0.42)
AcDXR2	EC:1.1.1.267	476	51.54	6.64	Chloroplast: very strong (0.96)
AcMCT	EC:2.7.7.60	319	35.52	6.78	Chloroplast, vacuole, cytoplasm, and plasma membrane: small (0.00)
AcHDS	EC:1.17.7.1; EC:1.17.7.3	740	82.27	6.32	Chloroplast: normal (0.72)
AcHDR	EC:1.17.7.4	460	51.91	5.29	Chloroplast: normal (0.73)
AACT1	EC: 2.3.1.9	408	41.85	6.67	Cytoplasm (also possible: peroxisome):normal (0.72)
AACT2	EC: 2.3.1.9	408	41.99	6.06	Cytoplasm: strong (0.90)
AACT3	EC: 2.3.1.9	412	42.72	8.47	Cytoplasm (also possible: peroxisome):normal (0.78)
AcHMGS1	EC: 2.3.3.10	458	50.78	6.25	Cytoplasm: normal (0.34)
AcHMGS2	EC: 2.3.3.10	458	50.63	6.32	Cytoplasm (also possible: plasma membrane):small (0.23)
AcHMGS3	EC: 2.3.3.10	291	32.87	9.17	Cytoplasm (also possible: nucleus): strong (0.82)
AcHMGR	EC: 1.1.1.34	580	62.2	7.16	ER, extracellular space, and Golgi apparatus: normal (0.34)
AcPMK	EC: 2.7.4.2	503	54.44	6.25	Extracellular space (also possible: plasma membrane and ER): small (0.22)
AcMDC	EC:4.1.1.33	421	46.57	6.63	Cytoplasm (also possible: nucleus): normal (0.56)
AcIDI	EC:5.3.3.2	289	32.99	4.99	Mitochondrion, nucleus, and cytoplasm:small (0.03)
AcFPPS	EC 2.5.1.10	342	39.23	5.31	Cytoplasm (also possible: nucleus): normal (0.68)
AcGGPS1	—	376	40.2	6.31	Chloroplast:very strong (1.00)
AcSSUII	—	331	36.2	5.93	Chloroplast: very strong (1.00)
AcPPS	EC 2.5.1.1	421	46.55	6.68	Mitochondrion:very strong (1.00)
AcSPS	—	426	46.72	4.94	Cytoplasm (also possible: chloroplast and mitochondrion): normal (0.58)
AcTPS1	—	587	67.4	5.45	Chloroplast: small (0.22)
AcSTS1	—	547	64.58	5.26	Cytoplasm: very strong (0.96)

AcSTS2	—	547	63.3	4.62	Cytoplasm: normal (0.56)
AcSTS3	EC:4.2.3.75	427	49.52	4.37	Cytoplasm: normal (0.36)

[a]The EC numbers for the enzymes encoded by the genes that searched out from the KEGG annotation file. The "—" represents undertermined enzyme number.

[b]The Yloc+ webserver is at https://abi-services.informatik.uni-tuebingen.de/yloc/webloc.cgi

Tab. S2 Sequence information on proteins used to construct the IDS phylogenetic tree

Organism[1]	Phylum	Genbank ID	Name in the phylogentic tree[2]
Synechocystis sp. PCC 6803	Cyanobacteria	WP_010871319.1	SyneGGPPS
Ostreococcus lucimarinus	Chlorophyta	XP_001420781.1	OlucGGPPS
Marchantia polymorpha	Bryophyta	PTQ43161.1	MpolGGPPS1
Marchantia polymorpha	Bryophyta	PTQ37335.1	MpolSSU
Physcomitrella patens	Bryophyta	XP_024402925.1	PpatGGPPS2
Physcomitrella patens	Bryophyta	XP_024359701.1	PpatGGPPS1
Abies grandis	Gymnospermae	AAL17614.2	AgraGGPPS1
Abies grandis	Gymnospermae	AAN01133.1	AgraGPPS1
Abies grandis	Gymnospermae	AAN01134.1	AgraGPPS2
Abies grandis	Gymnospermae	AAN01135.1	AgraGPPS3
Picea abies	Gymnospermae	ACZ57571.1	PabiGPPS1 (PaIDS1)
Picea abies	Gymnospermae	ACA21458.2	PabiGPPS2 (PaIDS2)
Picea abies	Gymnospermae	ACA21461.1	PabiGGPPS2 (PaIDS5)
Picea abies	Gymnospermae	ACA21462.1	PabiGGPPS1 (PaIDS6)
Picea sitchensis	Gymnospermae	ACN40538.1	PsitGGPPS1
Picea sitchensis	Gymnospermae	ACN40956.1	PsitGPPS1
Picea sitchensis	Gymnospermae	ACN40295.1	PsitGPPS2
Picea sitchensis	Gymnospermae	ACN40123.1	PsitSSUII
Amborella trichopoda	Angiospermae	XP_006856098.2	AtriGGPPS2
Amborella trichopoda	Angiospermae	XP_006853583.3	AtriGGPPS1
Amborella trichopoda	Angiospermae	XP_006828874.1	AtriSSUII
Amborella trichopoda	Angiospermae	XP_020521057.1	AtriSSUI
Antirrhinum majus	Angiospermae	AAS82860	AmajGGPPS1 (AmGPPS.LSU)
Antirrhinum majus	Angiospermae	AAS82859	AmajSSUI (AmGPPS.SSU)

Arabidopsis thaliana	Angiospermae	NP_195399	AthaGGPPS11
Arabidopsis thaliana	Angiospermae	NP_195558.1	AthaSSUII
Arabidopsis thaliana	Angiospermae	NP_175376.1	AthaGGPPS1
Arabidopsis thaliana	Angiospermae	NP_179452.1	AthaGGPPS2
Arabidopsis thaliana	Angiospermae	NP_179454.1	AthaGGPPS3
Arabidopsis thaliana	Angiospermae	NP_179960.1	AthaGGPPS4
Arabidopsis thaliana	Angiospermae	NP_188071.1	AthaGFPPS1 (AtGGPPS6)
Arabidopsis thaliana	Angiospermae	NP_001319550.1	AthaGFPPS2 (AtGGPPS7)
Arabidopsis thaliana	Angiospermae	NP_188651.1	AthaGGPPS8
Arabidopsis thaliana	Angiospermae	NP_189589.1	AthaGFPPS3 (AtGGPPS9)
Arabidopsis thaliana	Angiospermae	NP_189747.1	AthaGFPPS4 (AtGGPPS10)
Catharanthus roseus	Angiospermae	AGL91645.1	CrosGGPPS1 (CrGPPS.LSU)
Catharanthus roseus	Angiospermae	AGL91648.1	CrosGGPPS2
Catharanthus roseus	Angiospermae	AGL91646.1	CrosSSUI (CrGPPS.SSU)
Humulus lupulus	Angiospermae	ACQ90681.1	HlupSSUI (HlGPPS.SSU)
Humulus lupulus	Angiospermae	ACQ90682.1	HlupGGPPS1 (HlGPPS.LSU)
Lavandula x *intermedia*	Angiospermae	QHN60320.1	LintGGPPS2 (LiGPPS.LSU)
Lavandula x *intermedia*	Angiospermae	AGH33891.1	LintSSUI (LiGPPS.SSUI)
Lavandula x *intermedia*	Angiospermae	QHN60319.1	LintSSUII (LiGPPS.SSUII)
Lavandula x *intermedia*	Angiospermae	QHN60321.1	LintGGPPS1
Leucosceptrum canum	Angiospermae	ALT16903.1	LcanGFPPS1 (LcGFDPS)
Leucosceptrum canum	Angiospermae	ALT16901.1	LcanGGPPS1 (LcGGDPS1)
Leucosceptrum canum	Angiospermae	ALT16902.1	LcanGGPPS2 (LcGGDPS2)
Leucosceptrum canum	Angiospermae	ALT16904.1	LcanGGPPS3 (LcGGDPS3)
Leucosceptrum canum	Angiospermae	ALT16905.1	LcanGGPPS4 (LcGGDPS4)
Mentha piperita	Angiospermae	AAF08793.1	MpipGGPPS1 (MpGPPS.LSU)

Mentha piperita	Angiospermae	AAF08792.1	MpipSSUI (MpGPPS.SSU)
Nicotiana tabacum	Angiospermae	ADD49734.1	NtabGGPPS1
Nicotiana tabacum	Angiospermae	ADD49735.1	NtabGGPPS2
Nicotiana tabacum	Angiospermae	NP_001312601	NtabGGPPS3
Nicotiana tabacum	Angiospermae	NP_001312600	NtabGGPPS4
Nicotiana tabacum	Angiospermae	NP_001312125	NtabSSUII.1
Nicotiana tabacum	Angiospermae	XP_016445326	NtabSSUII.2
Nicotiana tabacum	Angiospermae	XP_016465609	NtabSSUI.1
Nicotiana tabacum	Angiospermae	XP_016455366	NtabSSUI.2
Oryza sativa	Angiospermae	XP_015647986	OsatGGPPS1
Oryza sativa	Angiospermae	XP_015614164	OsatGPPS1
Oryza sativa	Angiospermae	XP_015626863	OsatSSUII (OsGRP)
Salvia miltiorrhiza	Angiospermae	ACR19637.1	SmilGGPPS1
Salvia miltiorrhiza	Angiospermae	AEZ55682.1	SmilGGPPS2
Salvia miltiorrhiza	Angiospermae	AEZ55683.1	SmilGGPPS3
Salvia miltiorrhiza	Angiospermae	AEZ55681.1	SmilGGPPS4 (SmGPPS.LSU)
Salvia miltiorrhiza	Angiospermae	AEZ55678.1	SmilSSUI (SmGPPS.SSUI)
Salvia miltiorrhiza	Angiospermae	AEZ55679.1	SmilSSUII.1 (SmGPPS.SSUI.1)
Salvia miltiorrhiza	Angiospermae	AEZ55680.1	SmilSSUII.2 (SmGPPS.SSUII.2)
Sinapis alba	Angiospermae	CAA67330.1	SalbGGPPS1
Atractylodes chinensis	Angiospermae		AcSSUII
Atractylodes chinensis	Angiospermae		AcGGPPS1

[1] Species with full genome sequences are shaded.

[2] Original names of previously reported proteins are provided in parentheses.

Tab. S3 Sequence amplification and real-time quantitative PCR primers for the predicated unigenes involved in terpenoid biosynthesis

Gene name	Unigene ID	qPCR-F	qPCR-R
AcDXS1	transcript_HQ_AL_transcript6929/f16p0/2564	caaacagatcggtggccctatg	tcctctgcctttctcggtgatg
AcDXS2	transcript_HQ_AL_transcript9155/f2p0/2377	ccacttctcaagaaaaaagttccatatcaaagc	tcctggtttatcgccggaaaaatcaa
AcDXR1	transcript_HQ_AL_transcript17046/f3p0/1785	gcattgaagcatccaaactggagc	gatagattgagggtgaataacgatatcaatgttg
AcDXR2	transcript_HQ_AL_transcript18843/f4p0/1668	agcatcctatctggccgtcg	gtgtatgatagattgagggtgaataatgatctcaatatca
AcMCT	transcript_HQ_AL_transcript26407/f9p0/1166	tacgaacatccccaattctcatctgtct	tgccctaaaagtggaaggtactgc
AcHDS	transcript_HQ_AL_transcript7470/f8p0/2507	tactccaccgtgatggctctg	aactcccatacttacatcaatcaaccttt
AcHDR	transcript_HQ_AL_transcript18745/f20p0/1611	cgagcaacacctcccaccta	taccctcactagaacatcttccacaacc
AACT1	transcript_HQ_AL_transcript17643/f2p0/1753	ccggaatccctaatacagtggtctct	accaccagccacaactatatcattttga
AACT2	transcript_HQ_AL_transcript21001/f6p0/1540	gcaggaattcctaatacagtggtcagc	taccgccagccacaactatatcatttatg
AACT3	transcript_HQ_AL_transcript21936/f7p0/1480	aattggtgatgaacaacttaatgctcatgg	caacctggactgacccatcctt
AcHMGS1	transcript_HQ_AL_transcript18159/f2p0/1705	agaagagctttgctagattagtgttcagt	gtacatgttgccaacttgcttgttg
AcHMGS2	transcript_HQ_AL_transcript17322/f11p0/1746	cgctacggattggttgtctcg	tcaggcccaatcagcatagcaaca
AcHMGS3	transcript_HQ_AL_transcript26405/f2p0/1175	ctttgctcgcttggtgtttgac	tgtacatgtttcccaattgcttggga
AcHMGR	transcript_HQ_AL_transcript9834/f2p0/2309	actcccccctaaacctacctcc	ccatttgttaggtagagcggaaggg
AcPMK	transcript_HQ_AL_transcript16316/f3p0/1828	agcaatcagcctagaaatcgtcaaag	cgaatcacctaaggtaactgcaaatacag
AcMDC	transcript_HQ_AL_transcript21140/f4p0/1535	cctttgtaccaccactaccgtct	cttgattttgataccttttcttctcgtcctct
AcIDI	transcript_HQ_AL_transcript27075/f2p0/1115	gctacctcccaattctcccttcac	catgaccaacaaccttgtcattctcatc
AcFPPS	transcript_HQ_AL_transcript23194/f2p0/1401	atgagcagcgatctgaagtccaaattta	gcaacttgtagctgtcaacaacagaga
AcGGPS1	transcript_HQ_AL_transcript23159/f8p0/1370	ggttcagacatgttccatgtttaatggat	caaagtttcttgttcttctttggcaatcaca
AcSSUII	transcript_HQ_AL_transcript23838/f2p0/1355	gcgggtgggagtgatgatg	acccacataactcttccccttcc
AcPPS	transcript_HQ_AL_transcript20037/f2p0/1618	ggttcagacatgttccatgtttaatggat	caaagtttcttgttcttctttggcaatcaca
AcSPS	transcript_HQ_AL_transcript17477/f3p0/1753	cggtttccttcaagaaattatgcaagaaaagtga	gctccttgattctttcttctcacttaaaattggtg
AcTPS1	transcript_HQ_AL_transcript13390/f2p0/1996	gtgggtgagcccctggttag	cgcgtatcatcatctttactgcttcctt
AcSTS1	transcript_HQ_AL_transcript13799/f4p0/1996	gctaaaagaagaagttagaaaagagttaactgcca	gaaaccaaagggaaacactttgtaggtt

AcSTS2	transcript_HQ_AL_transcript15321/f5p0/1891	ctcttatcaaagctgctgctgctg	gcacataagagactctcggacgat
AcSTS3	transcript_HQ_AL_transcript10911/f5p0/2163	cccaaatacaagaagcattgacacaaccta	ctccttcttgtgcaatgattgaagca

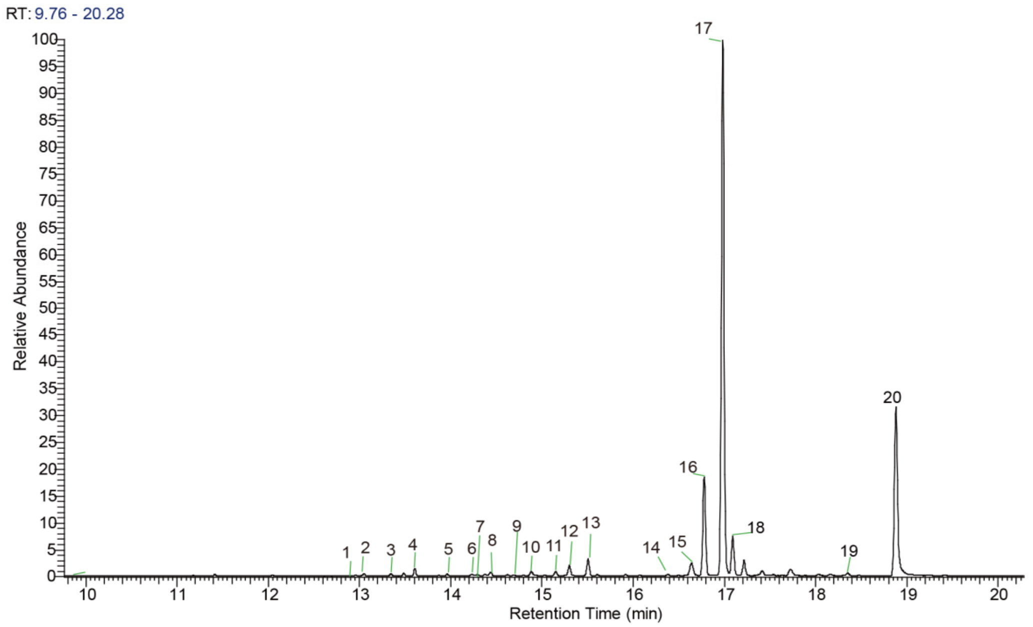

Fig. S1. Total ion flow diagram of GC-MS for essential oil extracted from the rhizome of *A. chinensis*. The X-axis of the graph shows the retention time. The chemicals that are identified as sesquiterpenes in the essential oil are labeled as chemicals 1-19, corresponding to the chemicals list in Table 1, in which the compound labeled as 16, 17, and 18 are identified as hinesol, β-eudesmol, and atractylon, respectively. The compound labeled as 20 is identified as atractylodin.

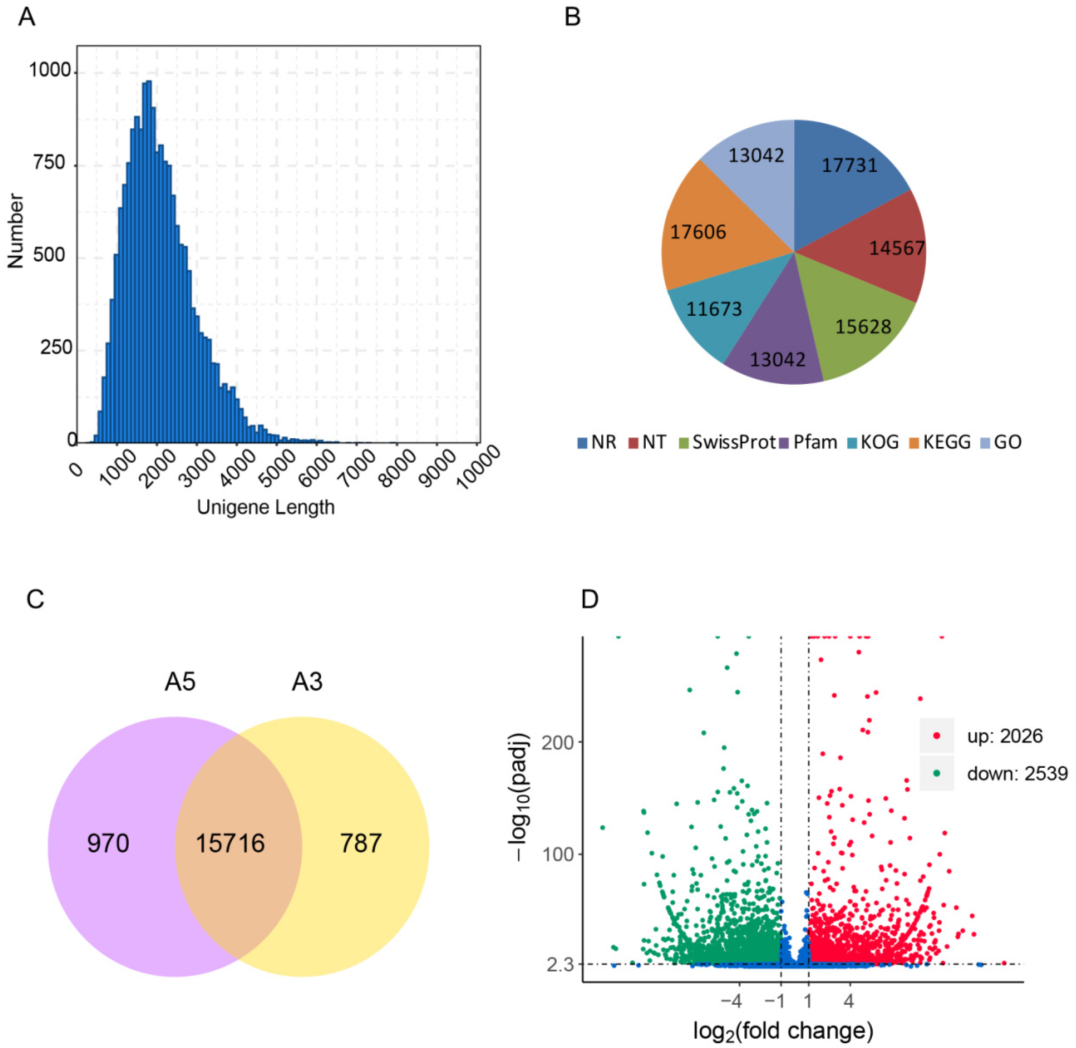

Fig. S2. Transcriptome analysis of the rhizomes of *A. chinensis*. (A) Numbers and length distribution of the unigenes. (B) Numbers of annotated unigenes in seven databases. (C) Numbers of genes expressed in three-year-old rhizome (A3) and five-year-old rhizome (A5). (D) The $\log_2$(fold change) on the horizontal ordinate represents the decreased fold changes of gene expression levels between A3 and A5. Padj represents the P-value and the ordinate on the vertical axis ($-\log_{10}$(padj)) represents the statistical significance of the gene expression changes. The scatterplot represents each unigene, where blue dots represent unigenes with no significant differences, red dots represent up-regulated unigenes with significant differences, and green dots represent down-regulated unigenes with significant differences.

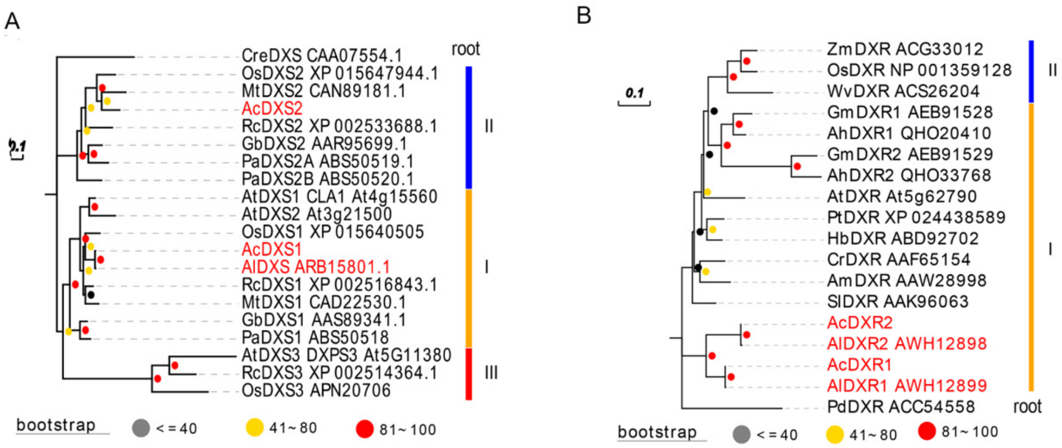

Fig. S3. Phylogenetic trees of plant DXS (A) and DXR (B) proteins. The accession numbers of plant DXSs and DXRs are displayed after their names. The abbreviated names of plant species are as follows: Cre, *Chlamydomonas reinhardtii*; Os, *Oryza sativa*; Mt, *Medicago truncatula*; At, *Arabidopsis thaliana*; Rc, *Ricinus communis*; Gb, Ginkgo; Pa, *Picea abies*; At, *Arabidopsis*; Rc, *Ricinus communis*; Pd, *Pinus densiflora*; Zm, Zea mays; Wv, *Wurfbainia villosa*; Gm, *Glycine max*; Ah, *Arachis hypogaea*; Pt, *Populus trichocarpa*; Hb, *Hevea brasiliensis*; Sl, *Solanum lycopersicum*; Am, *Antirrhinum majus*; Cr, *Catharanthus roseus*; Pp, *Prymnesium parvum*; and Ac and Al represent *Atractylodes chinensis* and *Atractylodes lancea*, respectively. Their deduced amino acid sequences were aligned using the MUSCLE program. The ML trees for DXS and DXR were constructed using IQ tree software with 1000 bootstrap replicates. The grey, orange, and red balls at the branches represent bootstrap value ranges of 0–40%, 41–80%, and 81–100%, respectively.

```
                           10        20        30        40        50        60        70        80        90       100
                  ....|....|....|....|....|....|....|....|....|....|....|....|....|....|....|....|....|....|....|....|
AcDXS1            MALSSFAFPGQVNQRSLTSHSPLFQHCLFGTDLQHPQSSHKPFNQASKRASGVRATLSERGEYYSQRPPTPLLDTINYPIHMKNLSIKELKQLADELRSD
AlDXS_ARB15801.1  .......L...L..................................................H.....................................

                          110       120       130       140       150       160       170       180       190       200
                  ....|....|....|....|....|....|....|....|....|....|....|....|....|....|....|....|....|....|....|
AcDXS1            VIFNVSKSGGHLGSNLGVVELTVALHYVFNTPQDKILWDVGHQSYPHKILTGRRDRMDTIRQTNGLAGFTKRSESEHDCFGTGHSSTTISAGLGMAVGRD
AlDXS_ARB15801.1  ....................................................................................................

                          210       220       230       240       250       260       270       280       290       300
                  ....|....|....|....|....|....|....|....|....|....|....|....|....|....|....|....|....|....|....|
AcDXS1            LKGGTNDVVAIIGDGAMTAGQAYEAMNNAGYLDSDMIVILNDNKQVSLPTATLDGPIPPVGALSSALSRLQSNRPLRELREVAKEVTKQIGGPMHEIAAK
AlDXS_ARB15801.1  ....................................................................................................

                          310       320       330       340       350       360       370       380       390       400
                  ....|....|....|....|....|....|....|....|....|....|....|....|....|....|....|....|....|....|....|
AcDXS1            VDEYARGMISGSGSSLFEELGLYYIGPVDGHSIDDLVAILKEVKSTKTTGPVLIHVITEKGRGYPYAEKAADKYHGVAKFDPATGKQFKSSAPTQSYTTY
AlDXS_ARB15801.1  ...............................N....................................R...............................

                          410       420       430       440       450       460       470       480       490       500
                  ....|....|....|....|....|....|....|....|....|....|....|....|....|....|....|....|....|....|....|
AcDXS1            FAEALIAEAEADKRIVSIHAAMGGGTGLNLFLRRFPSRCFDVGIAEQHAVTFAAGLACEGLKPFCAIYSSFLQRGYDQVVHDVDLQKLPVRFALDRAGLV
AlDXS_ARB15801.1  ..........V.....G...................................................................................

                          510       520       530       540       550       560       570       580       590       600
                  ....|....|....|....|....|....|....|....|....|....|....|....|....|....|....|....|....|....|....|
AcDXS1            GADGPTHSGSFDVTYMACLPNMVVMAPSDEAELFHMVATAAAIDDRPSCFRYPRGNGIGMPLPPGNKGIPLEIGKGRIMIEGQRVALLGYGTAVQSCLAA
AlDXS_ARB15801.1  ....................................................................................................

                          610       620       630       640       650       660       670       680       690       700
                  ....|....|....|....|....|....|....|....|....|....|....|....|....|....|....|....|....|....|....|
AcDXS1            AGLVQQRGLNITVADARFCKPLDHTLIRTLAKSHEVLITVEEGSIGGFGSHVAHFMALDGLLDGNLKWRPLVLPDRYIDHGAPADQLAEAGLTPSHIAAT
AlDXS_ARB15801.1  ..............................................................................E.....................

                          710
                  ....|....|....|.
AcDXS1            VFNVLGQTREALEVMS
AlDXS_ARB15801.1  ................
```

Fig. S4. Sequence alignment of AcDXS1 and AlDXS (ARB15801.1).

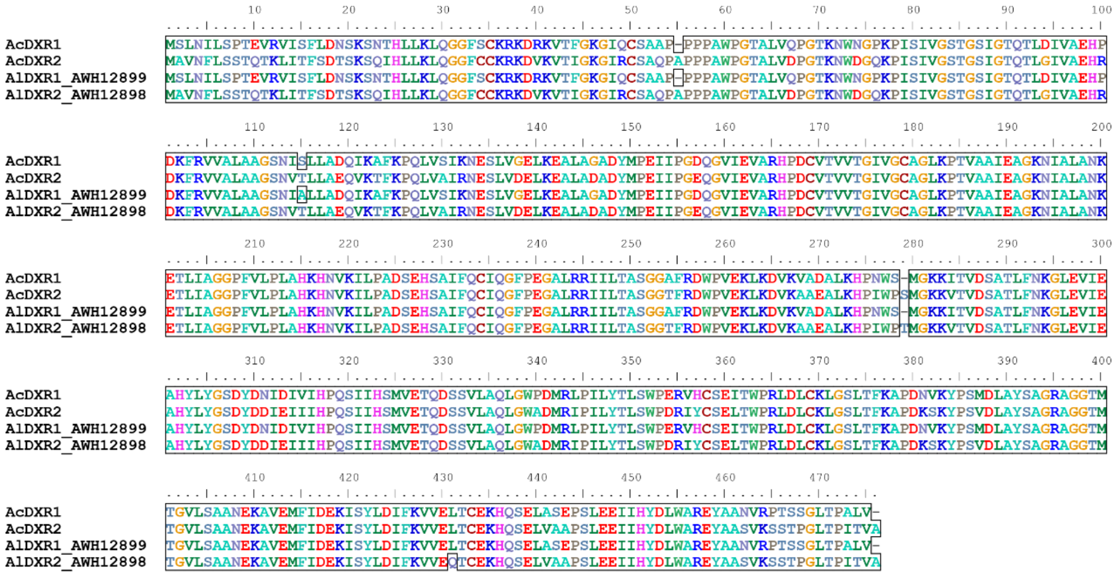

Fig. S5. Sequence alignment of AcDXR and AlDXR proteins.

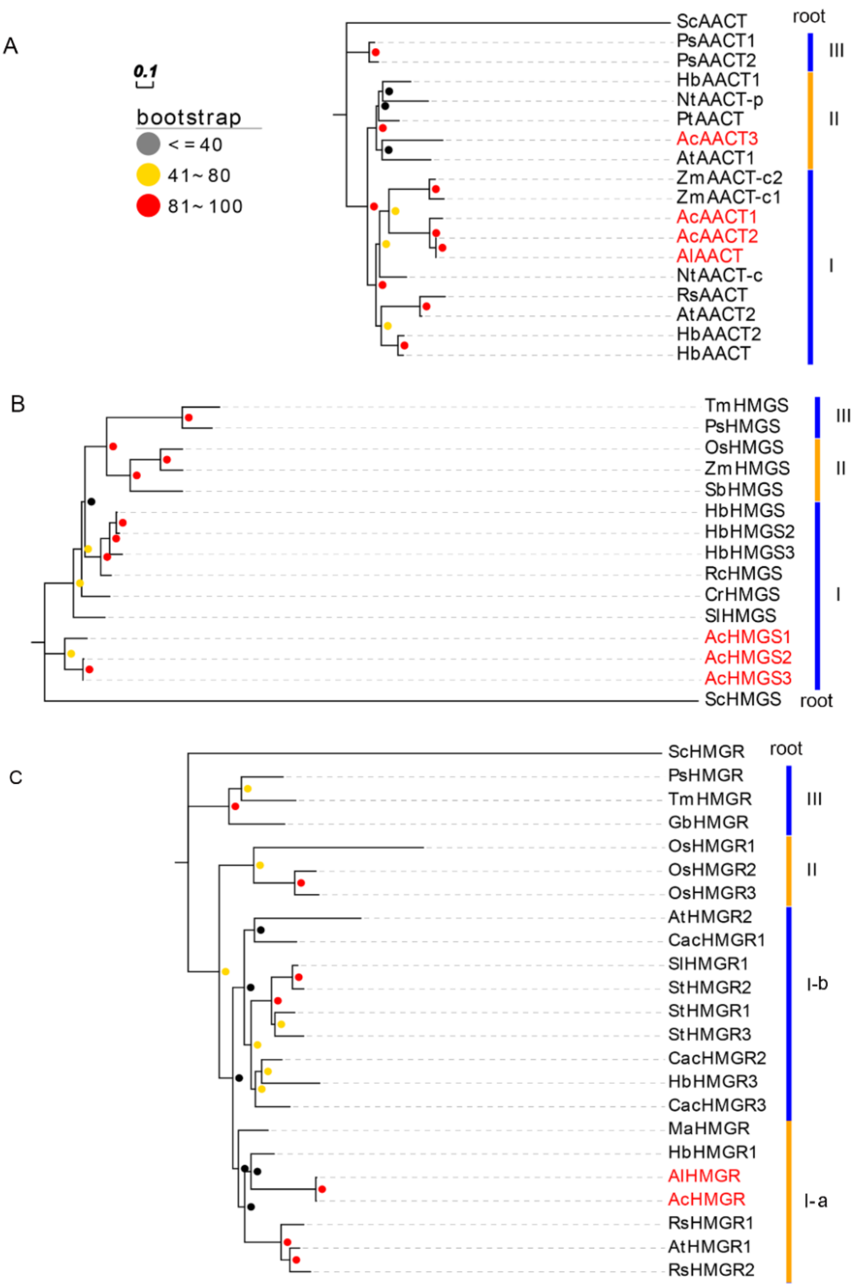

Fig. S6. Rooted ML phylogenetic trees of plant AACT (A), HMGS (B), and HMGR proteins. The ML trees were constructed using IQ tree software with 1000 bootstrap replicates. The grey, orange, and red balls at the branches represent bootstrap value ranges of 0–40%, 41–80%, and 81–100%, respectively. The abbreviated names of species are as follows: Sc, *Saccharomyces cerevisiae*; Ps, *Pinus*; Hb, *Hevea brasiliensis*; Nt, *Nicotiana tabacum*; Pt, *Populus trichocarpa*; At, *Arabidopsis*; Zm, *Zea mays*; Rs, *Raphanus sativus*; Hb, *Hevea brasiliensis*; Tm, *Taxus x media*; Os, *Oryza sativa*; Sb: *Sorghum bicolor*; Rc, *Ricinus communis*; Cr, *Catharanthus roseus*; Sl, *Solanum lycopersicum*; Gb, *Ginkgo biloba*; Ca, *Camptotheca acuminate*; St, *Solanum tuberosum*; Ma, *Morus alba*; Rs, *Raphanus sativa*; Ac and Al (with red type) represent *Atractylodes chinensis* and *Atractylodes lancea*, respectively. The sequence accession numbers of proteins were: ScAACT, P41338.3; PsAACT1, ACN40771.1; PsAACT2, ACN41149.1; HbAACT1, AF429383_1; HbAACT2, BAF98277.1; HbAACT, BAF98276.1; NtAACT-c, AAU95618.1; NtAACT-p, AAU95619.1; PtAACT, XP_002308755.1; ZmAACT-c1,

NP_001148667.1; ZmAACT-c2, ACG34735; AtAACT1, At5g47720; AtAACT2, At5g48230; RsAACT, CAA55006.1; AlAACT, QLM00050; AcAACT1, this study; AcAACT2, this study; AcAACT3, this study; TmHMGS, AAT73206.1; PsHMGS, CAA65250.1; OsHMGS, Os08g0544900; ZmHMGS, ACG33137.1; SbHMGS, XP_002468628; HbHMGS, BAF98279.1; HbHMGS2, AAS46245.1; HbHMGS3, AF429389_1; RcHMGS, XP_002509692.1; CrHMGS, AEC13715.1; SlHMGS, ABX55778.1; ScHMGS, P54839; AcHMGS1, this study; AcHMGS2, this study; AcHMGS3, this study; ScHMGR, AAA34676.1; PsHMGR, ACN40476.1; TmHMGR, AAQ82685.1; GbHMGR, AAU89123.1; OsHMGR1, AAA21720; OsHMGR2, AAD08820; OsHMGR3, AF110382; AtHMGR1, AT1G76490.1; AtHMGR2, AT2G17370.1; CacHMGR1, AAA33040.1; CacHMGR2, AAB69727.1; CacHMGR3, AAB69726.1; SlHMGR1, AAA34169.1; StHMGR1, AAA93498.1; StHMGR2, AAB52551.1; StHMGR3, AAB52552.1; HbHMGR1, CAA38469.1; HbHMGR3, AAA33360.1; MaHMGR, AAD03789.1; RsHMGR1, CAA48610.1; RsHMGR2, CAA48611.1; AlHMGR, AWH12900.1; AcHMGR, this study.

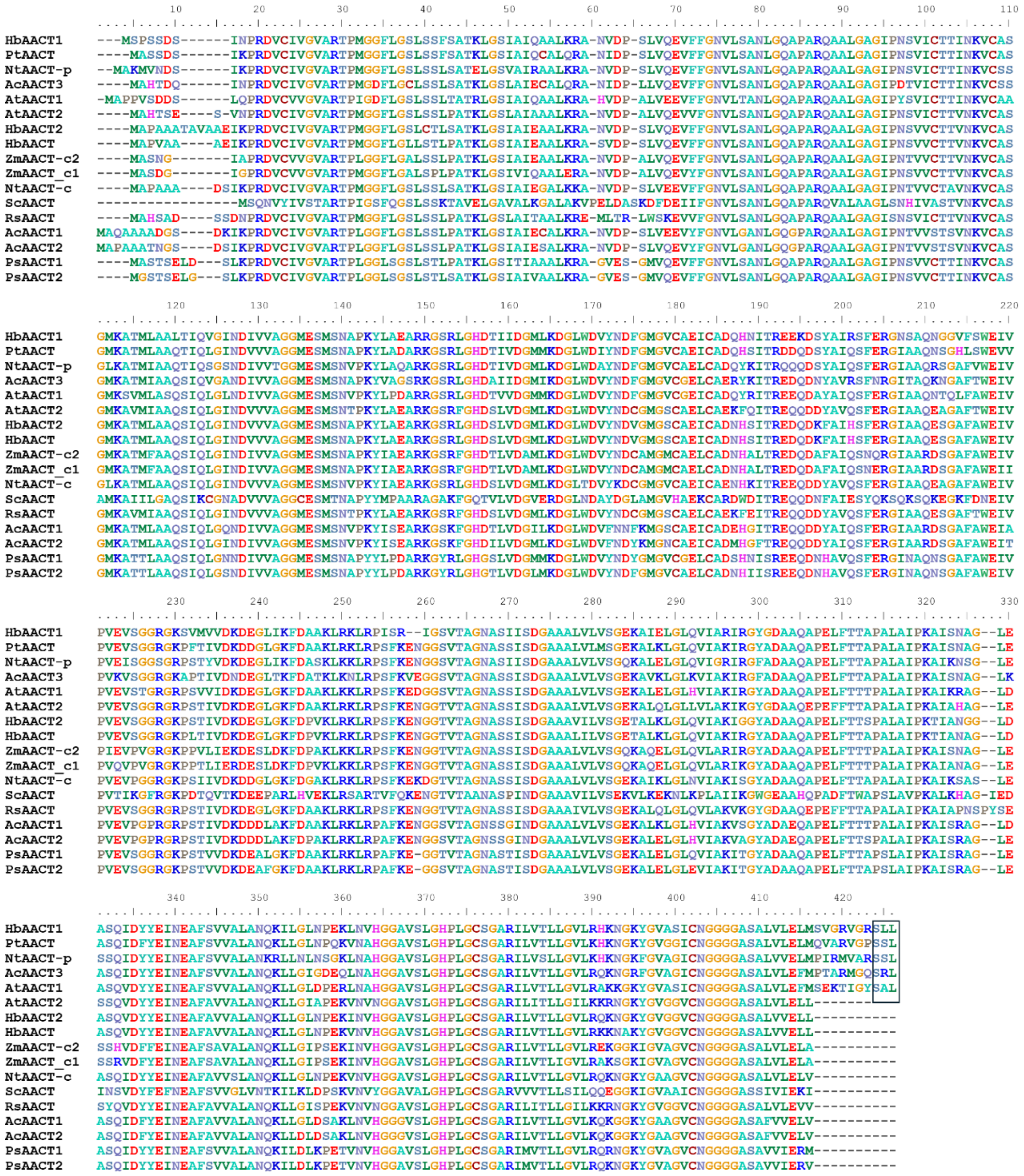

Fig. S7. Sequence alignment of plant AACT proteins.

```
                              10        20        30        40        50        60        70
                      ....|....|....|....|....|....|....|....|....|....|....|....|....|....|
AlHMGR [A. lancea]    MDIRRRLPPKPTSGTLVNHQKPVTNDDNRSSSSRSNGVVDRSPSPVPKASDALPLPLYLTNGIFFTLFFC
AcHMGR [A. chinensis] ................L.....................................................

                              80        90       100       110       120       130       140
                      ....|....|....|....|....|....|....|....|....|....|....|....|....|....|
AlHMGR [A. lancea]    VAYYLLHRWRDKIRSSTPLHVVTLSELAAIVSLIASFIYLLGFFGIDFVQSFITTARGEEDNFVLEGEVE
AcHMGR [A. chinensis] ......................................................................

                             150       160       170       180       190       200       210
                      ....|....|....|....|....|....|....|....|....|....|....|....|....|....|
AlHMGR [A. lancea]    HHSETKPRIMDPIPSIVLTSIEDEEIVKSVVCGSVPSYSLESKLGDCLRAASIRRQALERTTRRSLLGLP
AcHMGR [A. chinensis] ......................................................................

                             220       230       240       250       260       270       280
                      ....|....|....|....|....|....|....|....|....|....|....|....|....|....|
AlHMGR [A. lancea]    LEGFDYESILGQCCEMPVGYVQLPVGIAGPLLLNGCEYMVPMATTEGCLVASTNRGCKAIFASGGATAIL
AcHMGR [A. chinensis] ....N.................................................................

                             290       300       310       320       330       340       350
                      ....|....|....|....|....|....|....|....|....|....|....|....|....|....|
AlHMGR [A. lancea]    LKDGMTRAPVVRFDTAERASQLKFFLEDAINFNTLSLIFNKSSRFARLQYIQCAIAGKNLYIRFTCSTGD
AcHMGR [A. chinensis] ......................................................................

                             360       370       380       390       400       410       420
                      ....|....|....|....|....|....|....|....|....|....|....|....|....|....|
AlHMGR [A. lancea]    AMGMNMVSKGVQNVLDFLQSDFPDMDVIGISGNFCSDKKPAAVNWIEGRGKSVVCEAVITEEVVKKVLKT
AcHMGR [A. chinensis] ...................N..................................................

                             430       440       450       460       470       480       490
                      ....|....|....|....|....|....|....|....|....|....|....|....|....|....|
AlHMGR [A. lancea]    TVPGLVELNMLKNLTGSAIAGSLGGFNAHAANIVSAVFIATGQDPAQNIESSHCLTMMEAVNNGKDPHIS
AcHMGR [A. chinensis] ..................................................................L...

                             500       510       520       530       540       550       560
                      ....|....|....|....|....|....|....|....|....|....|....|....|....|....|
AlHMGR [A. lancea]    VTMPSIEVGTVGGGTQLASQAACLNLLGAKGANEESPGSNARLLASIVAGSVLAGELSLMSAIAAGQLVK
AcHMGR [A. chinensis] ......................................................................

                             570       580
                      ....|....|....|....|..
AlHMGR [A. lancea]    SHMKYNRSNRDMAAIASASASS
AcHMGR [A. chinensis] ...................S--
```

Fig. S8. Sequence alignment of HMGR proteins from *A. lancea* and *A. chinensis*.

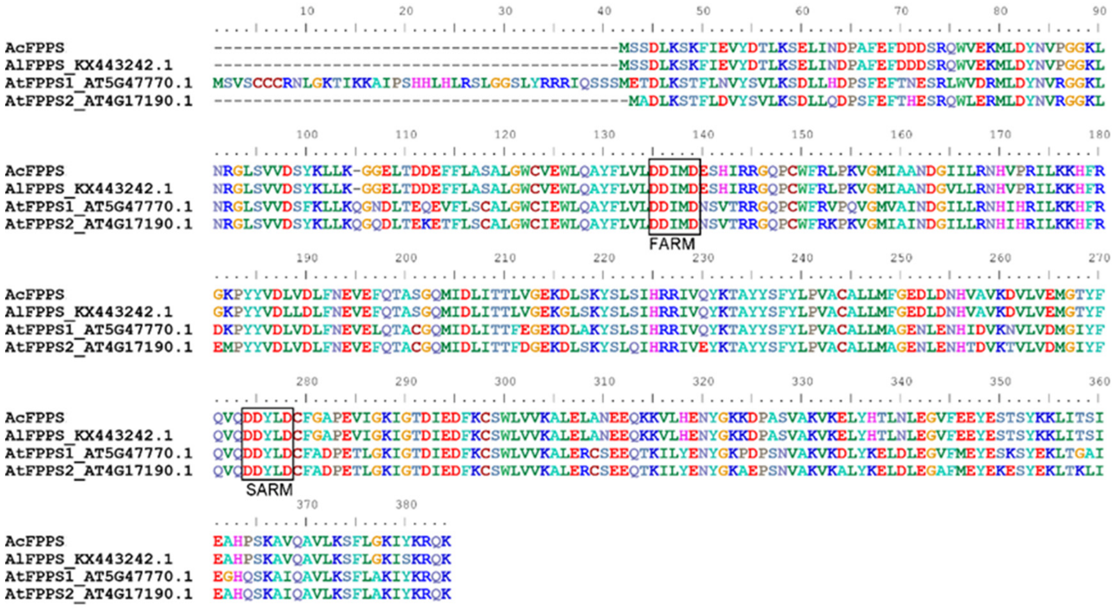

Fig. S9. Sequence alignment of FPPS from *Arabidopsis*, *A. chinensis*, and *A. lancea*.

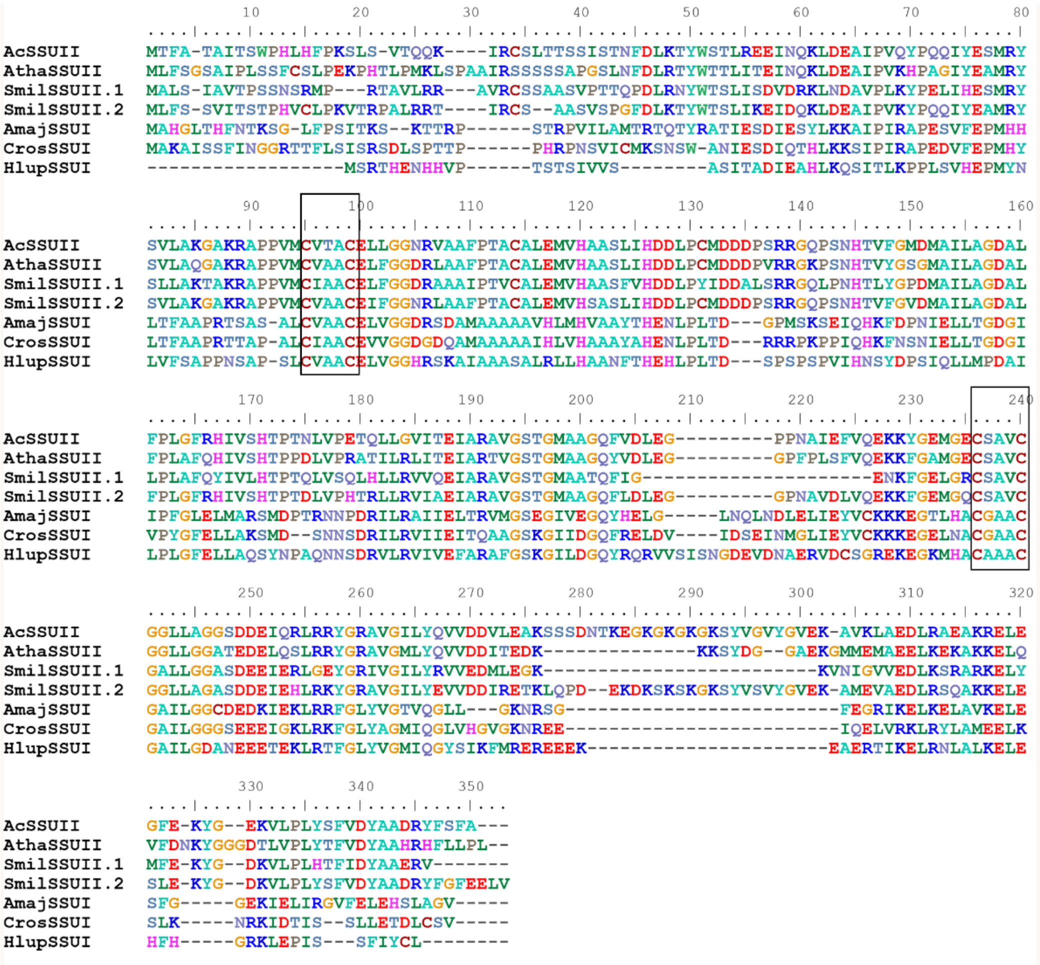

Fig. S10. Sequence alignment of the small subunit of heterodimeric IDS proteins (SSU) from various species, such as *Arabidopsis* (Atha), *A. chinensis* (Ac), and *Antirrhinum majus* (Amaj). The sequence details and accession numbers are shown in Tab. S2.

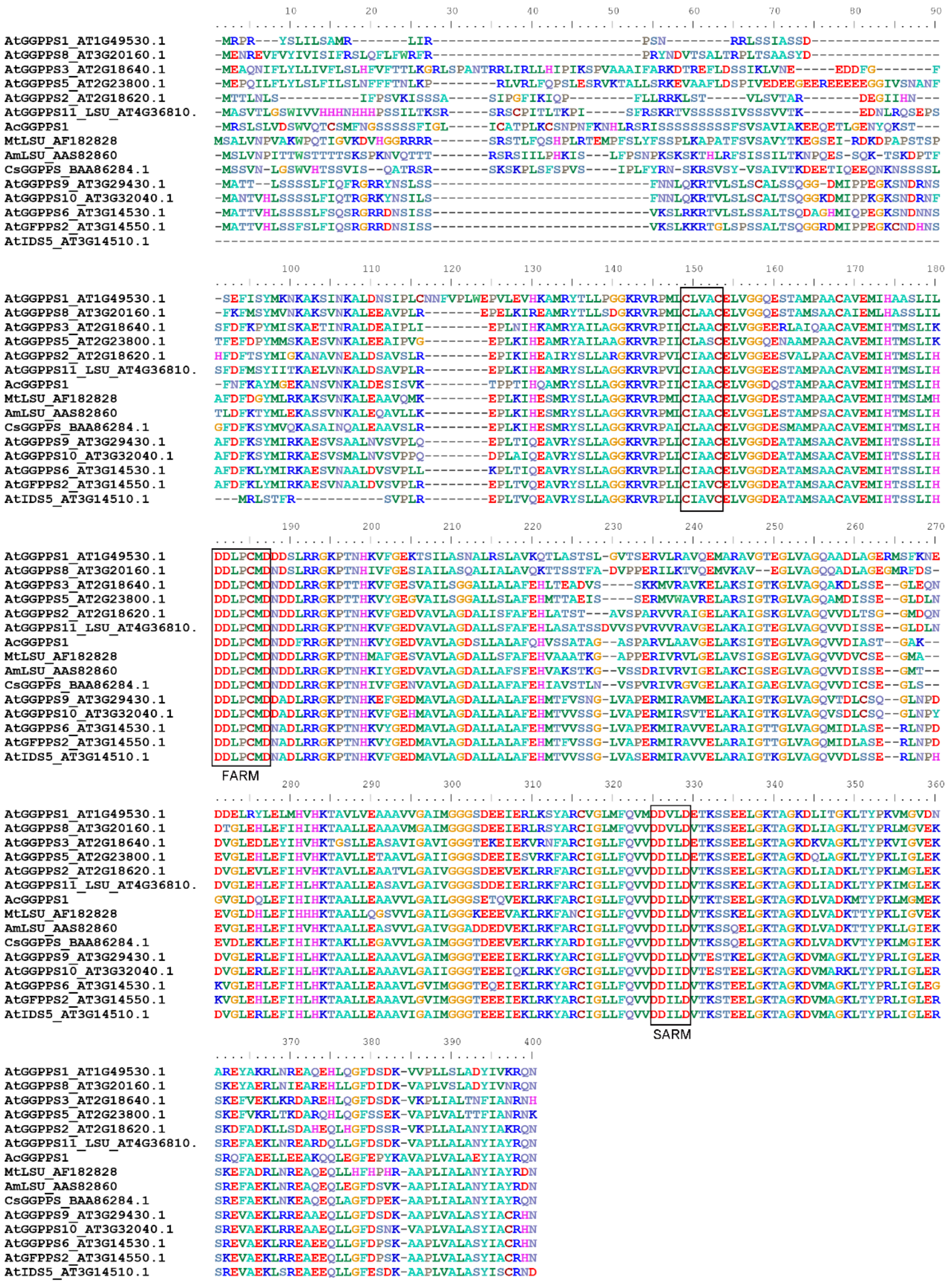

Fig. S11. Sequence alignment of GGPPS or GFPPS proteins from *Arabidopsis*, *A. chinensis*, peppermint, *Antirrhinum majus* and *Croton sublyratus*.

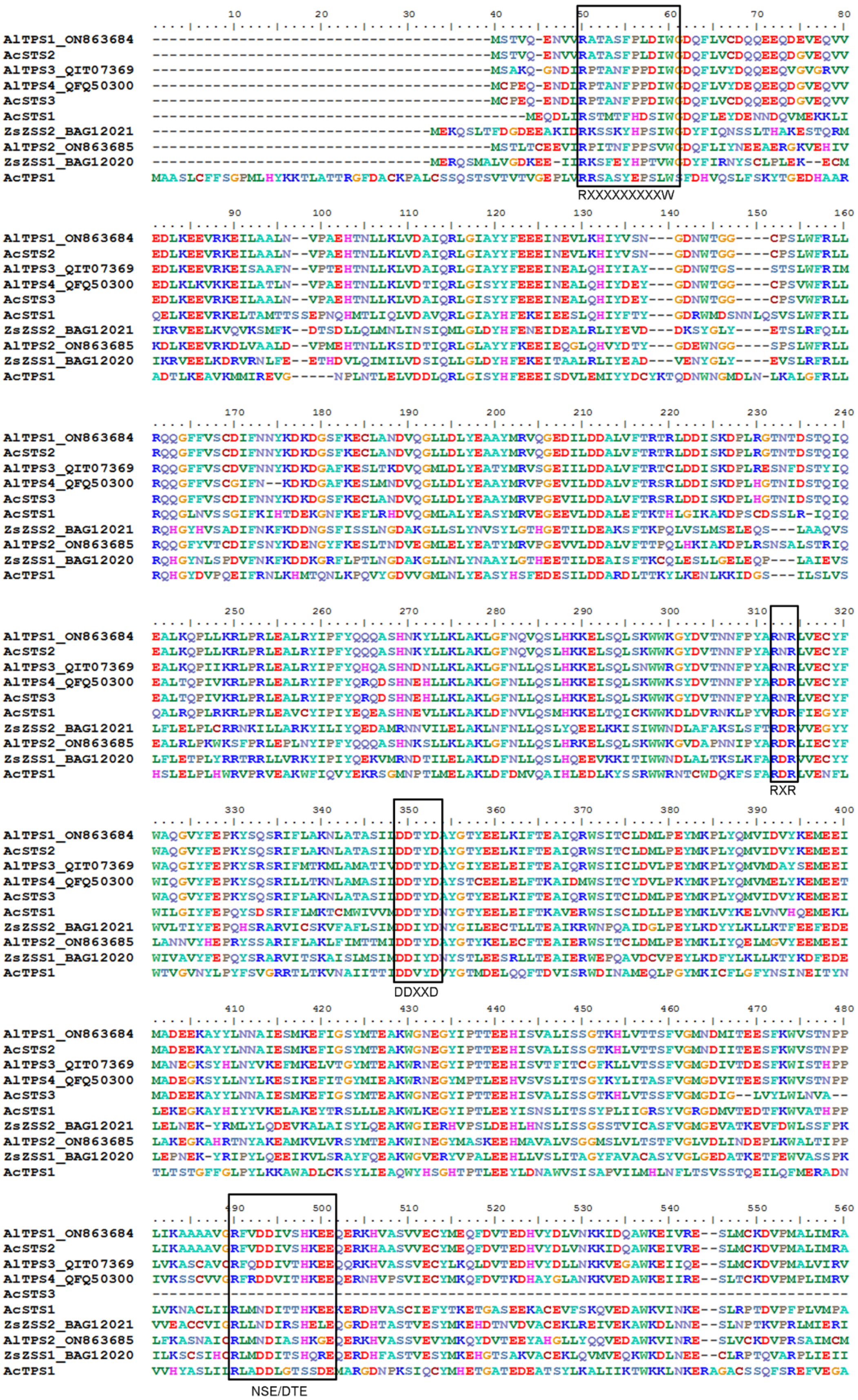

Fig. S12. Sequence alignment of terpene synthase from *A. lancea* (Al-), *A. chinensis* (Ac-) and *Zingiber zerumbet* Smith (Zs-).
